# The electrophysiology of ventricular cell pairs

**DOI:** 10.1007/s00424-025-03132-1

**Published:** 2025-12-25

**Authors:** Massimiliano Zaniboni

**Affiliations:** Department of Chemistry, Life Sciences and Environmental Sustainability, University of Parma (Italy), Parco Area delle Scienze 11/A, Parma, 43100 Italy

**Keywords:** Ventricular cell pairs, Source-sink properties, Uni-directional block, Gap junctional resistance, Purkinje-ventricular junction, Ventricular-fibroblast coupling

## Abstract

Modern cardiac electrophysiology is largely focused on global electrical properties of tissue, frequently assessed by extracellular electrical recordings or optical mapping, whereas studies on the microscopic nature of action potential conduction are more rare, though fundamental to understand the whole-heart behavior. The electrophysiological dynamics of two ventricular myocytes electrically connected via gap junctions is the simplest conceivable model to study the laws governing the conduction of the electrical impulse within the heart ventricles. Since the enzymatic dispersion of cardiac cells has made it possible to isolate and investigate cardiac cell pairs directly, additional techniques have been introduced to investigate the problem, including coupling clamp, dynamic clamp, and numerical simulations. The physiology of a ventricular cell pair allows to define the basic laws governing the electrotonic interaction between cardiac cells in the absence of factors, like fiber orientation, heterogeneous spatial dispersion of electrophysiological properties, complex interaction with vessels and fibrotic tissue, that play a significant role in multicellular tissue studies. However, these laws remain at the base of our understanding of cardiac electrical functioning and should be bear in mind when dealing with the complexity of the whole organ level. This review will face both the technical issues encountered with the studies of ventricular cell pairs as well as the results found by the different authors concerning the physiological electrical coupling between cells, their physiological and pharmacological modulation, and the complex interactions between the conducted action potentials in the two cells.

**Supplementary Information:**

The online version contains supplementary material available at 10.1007/s00424-025-03132-1.

## Introduction

**Fig. 1:**
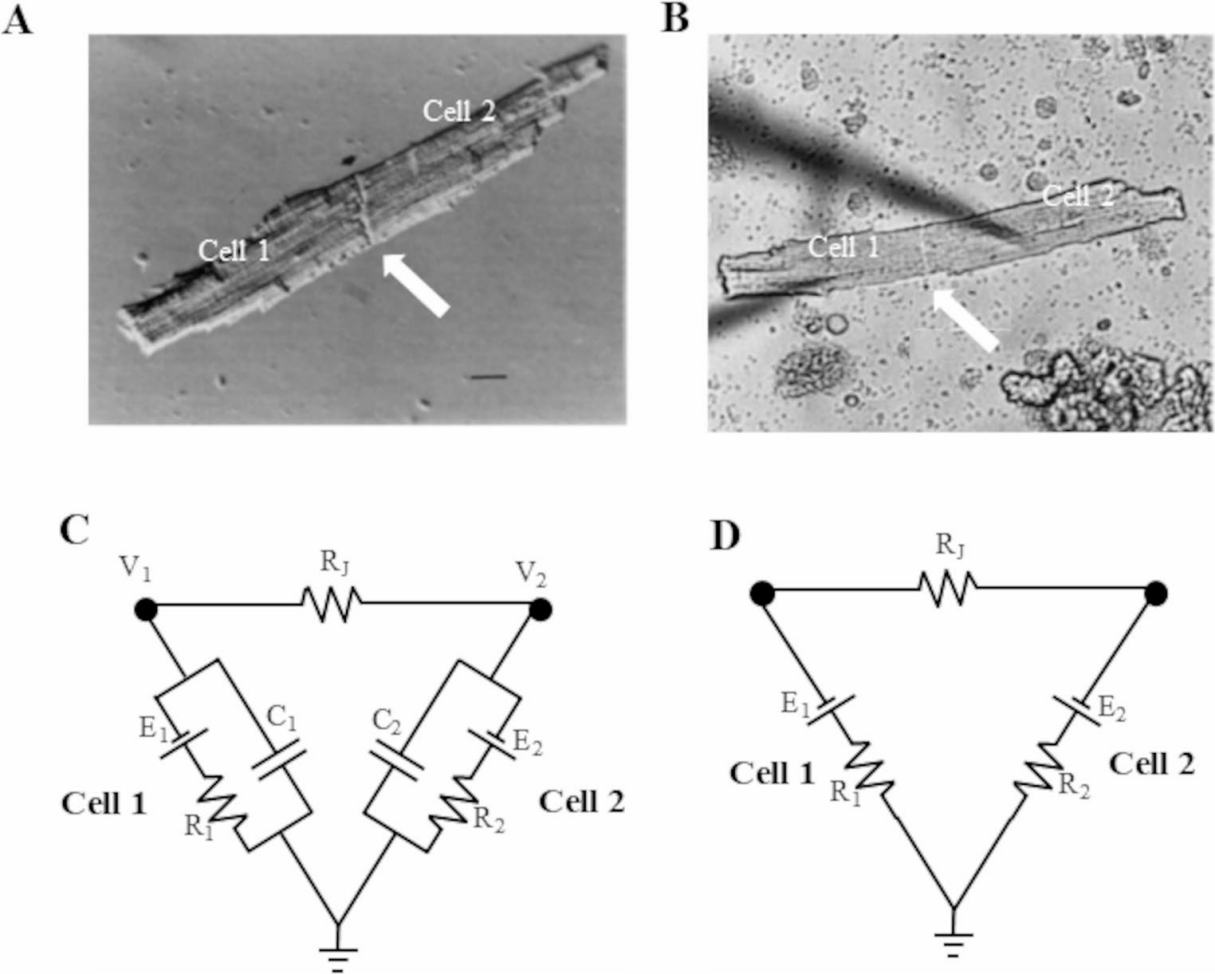
Cell pairs and their equivalent circuit. After enzymatic dispersion, cell pairs can be found (**A**) (from [37] and braught simultaneously in patch clamp whole cell configuration (**B**, the shades of the two patch pipettes are visibile) (from Zaniboni et al. 2006). White arrows points to the gap junctional region. The equivalent electrical circuit of the cell pair forms the so-called *delta circuit* (**C**), which appears simplified (**D**) when considered in steady state conditions

The cardiac tissue is largely made of excitable cells electrically connected to each other’s via gap junctions. Most of the studies on action potential (AP) propagation and on the intercellular electrical coupling have been, and still are, conducted on multicellular preparations from various regions of the heart by focusing on parameters related to the combination of cellular and intercellular electrical properties into the complex three-dimensional structure of the tissue, which include fiber orientation, vessels, fibroblasts and heterogeneous spatial distribution of electrophysiological properties. Techniques of enzymatic dispersion of cardiac myocytes have made it possible to access these properties at the level of isolated cell pairs. Among the yield of single cells resulting from enzymatic dispersion, cell pairs physically connected by functional gap junctions can be found (Fig. 1A) and analyzed, providing the simplest possible experimental model to clarify the dynamics of electrical communication between cardiac cells. This review will focus primarily on studies concerning this model, or models that are equivalent from the electrical point of view. Given the vastity of the literature involved, I will focus here only on ventricular cell pairs.

I will describe the different techniques used to access the electrophysiology of ventricular cell pairs, which include the double-patch clamp, the coupling-clamp, the dynamic clamp techniques, as well as the numerical simulations of cell pairs. The equivalent electrical circuit of a cell pair is essentially made by a simple triangular combination, it is also called “delta circuit” (Fig. 1C and D), of three resistors, two representing the membrane resistance of the two cells of the pair, and one the electrical resistance of the gap junctional coupling between the two. Of the three nodes of the circuit, two represent the membrane voltage of each cell and one the ground. Based on this equivalent circuit, I will describe the electrophysiological protocols adopted to measure the three resistances, and particularly the gap junctional resistance. I will also describe the basic laws governing the relationship between the membrane voltages of the two cells when electrically connected via the gap junctions, first in the simpler case of subthreshold potentials and then during AP propagation. Results obtained with double patch clamp, coupling clamp, dynamic clamp, and computer simulations will be reviewed.

## Methodological section

### The double-patch clamp technique

**Fig. 2:**
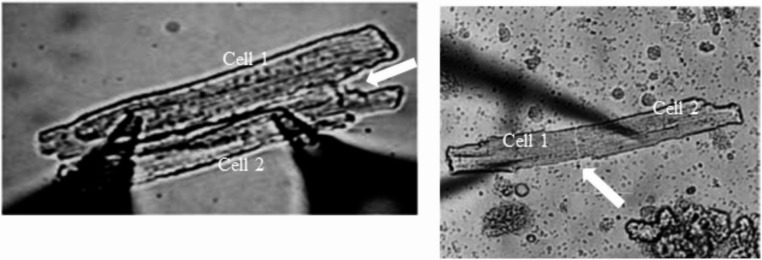
Pairs configurations. In the yeald of enzymatically dispersed cells, cell pairs can be found, which are coupled by gap junctions longitudinally (side-by-side, on the left) (from [68]) or at their extremes (end-to-end, on the right) (from [97])

A pair of cells electrically coupled by gap junctions can be brought simultaneously in patch clamp whole cell configuration (Fig. 1A and B) and described by the equivalent circuit of Fig. 1C. Cell pairs can be found in side-by-side or in end-to-end configuration (Fig. 2), which are described by the same circuit. The model is based on the mutual relationship between the three resistive elements R_1_, R_2_, R_j_, described above. The membranes of the two cells comprehend also the corresponding electrical capacitances (C_1_ and C_2_) in parallel with their resistances. Finally, in series of each resistance, the corresponding electromotive force (E_1_ and E_2_) is inserted, which reflects the Nernst equilibrium potential of the permeant ions and, in uncoupled conditions, represents the intrinsic resting membrane potential of the two cells. V_1_ and V_2_ are the membrane potential values read by the patch clamp amplifiers. Near resting potential conditions, i.e. for deflections of V_1_ and V_2_ of only few mV around their resting values, the main contribution to the membrane resistance is that of I_K1_ current [11], whose resistance is nearly constant in that voltage range, making the study of the equivalent circuit straightforward. Also, in steady state condition, i.e. when V_1_ and V_2_ do not vary in time, the capacitive component of the membrane current is zero and the equivalent circuit can be furtherly simplified to that of Fig. 1D. Finally, when V_1_ and/or V_2_ reach the threshold for excitation, the membrane resistive component of the equivalent circuit must be replaced by the parallel combination of all the ion currents (membrane resistances) that contribute to the AP, each one in series with the corresponding electromotive force of the reversal potential associated to the permeant ionic species. Thus, the validity of the equivalent circuit, as it is represented in Fig. 1C and D, is restricted to cells at their resting potential or for membrane potential changes that do not reach the threshold for excitation.

### The coupling-clamp technique

**Fig. 3:**
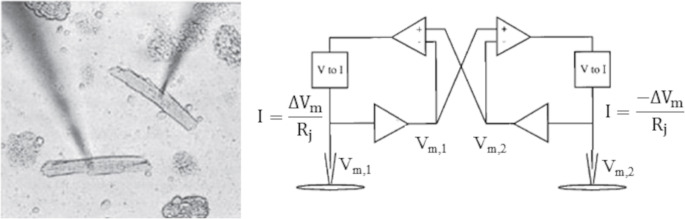
The coupling-clamp technique. Two separate cells are brought simultaneously in patch clamp whole cell configuration (left panel, Zaniboni, unpublished results) and the membrane potentials recorded by the patch amplifiers are sent to additional amplifiers (right panel) to compute the voltage difference (ΔV_m_). The outputs are sent to voltage-current converters that supply equal and opposite current into the two cells, exactly as they were directly connected with an electrical resistance R_j_, which can thus be chosen arbitrarily by varying the gain of the amplifiers. The equivalent electrical circuit of two coupling-clamped cells is thus identical to that reported in Fig. 1 (modified by [25]

Whereas with the double-patch clamp technique the R_j_ is that, constant, of each given cell pair, an experimental approach has been developed that allows the electrical coupling of physically isolated cells via an arbitrarily chosen R_j_, so-called coupling-clamp technique [71]. Two separated cells are brought simultaneously in patch clamp whole cell configuration and their electrodes connected via an electronic circuit that simulates the existence of a given, arbitrarily set, junctional resistance R_j_ between them (see details in Fig. 3). Through this experimental configuration, also described by the equivalent electrical circuit of Fig. 1, the electrical coupling between pairs made of the same cell types or of different cell types can be studied, including cases in which one of the two cells of the pair is not excitable, like a fibroblast. Also, since, as mentioned above, resting cardiac cells behave linearly, i.e. ohmically, in the restricted voltage range (approximately ± 10 mV) around the resting potential, one of the two cells can be replaced by an electrical parallel RC circuit where an electromotive force is introduced in series to the R element to confer the desired resting membrane potential.

### The dynamic clamp technique

It is an evolution of the coupling clamp technique that allows, by adopting ‘‘hard’’ real-time operating systems [20], to couple real cells to numerically reconstructed action potentials of any kind. Thus, cell pairs made, for instance, of a real rat left ventricular cell and the numerical model of the same cell type can be achieved, or cell pairs made of a real ventricular cell with the numerical model of a myofibroblast, etc.

### Numerically simulated cell pairs

The numerical simulation of a single cell AP consists in solving simultaneously the differential equations of the Hodgkin-Huxley type for the membrane potential and for the gating variables (and for carriers, pumps, etc.) reconstructed from voltage clamp experiments on the cell type to be described [39]. Thus, if we omit for brevity the differential equations of the gating variables, the numerical reconstruction of a cellular AP consists in solving the following first order differential equation:

1 $$\:\:{C}_{m}\:\frac{d\:{V}_{m}}{dt}\:+\:\sum\limits_{i=1}^{n}{I}_{ion,i}\:+\:{I}_{stim}=0$$

with C_m_ the electrical membrane capacitance, V_m_ the membrane potential, I_ion, i_ the ion currents, I_stim_ the stimulus current. To reconstruct the electrotonic interaction between two cells connected by a resistance R_j_, the following system must be solved, where the equations of the two single cells are coupled by the terms on the left of each equation, that simply represent the electrotonic current flowing between the two.

2 $$\:\left\{\begin{array}{c}\frac{{V}_{1}-\:{V}_{2}}{{R}_{j}}\:=\:{C}_{m}\:\frac{d\:{V}_{1}}{dt}\:+\:\sum\limits_{i=1}^{n}{I}_{ion1,i}\:+\:{I}_{stim,1}\\\:\frac{{V}_{2}-\:{V}_{1}}{{R}_{j}}\:=\:{C}_{m}\:\frac{d\:{V}_{2}}{dt}\:+\:\sum\limits_{i=1}^{n}{I}_{ion2,i}+\:{I}_{stim,2}\end{array}\right.$$

Hodgkin-Huxley type equations like (1) or (2) are solved numerically, i.e. by discretizing time and obtaining, for each time step, the updated vector containing all the differentiated variables, by means of Euler - types methods.

### The measure of R_j_

When both cells of a physically connected cell pair are either impaled with standard microelectrodes or brought in patch clamp whole cell configuration, the electrical equivalence of Fig. 1C is established and R_1_, R_2_, and R_J_ can either be measured in current clamp or voltage clamp mode. As mentioned above, in steady state conditions we refer to the equivalent circuit of Fig. 1D.

#### The measure of gap junctional electrical coupling in current clamp mode

**Fig. 4:**
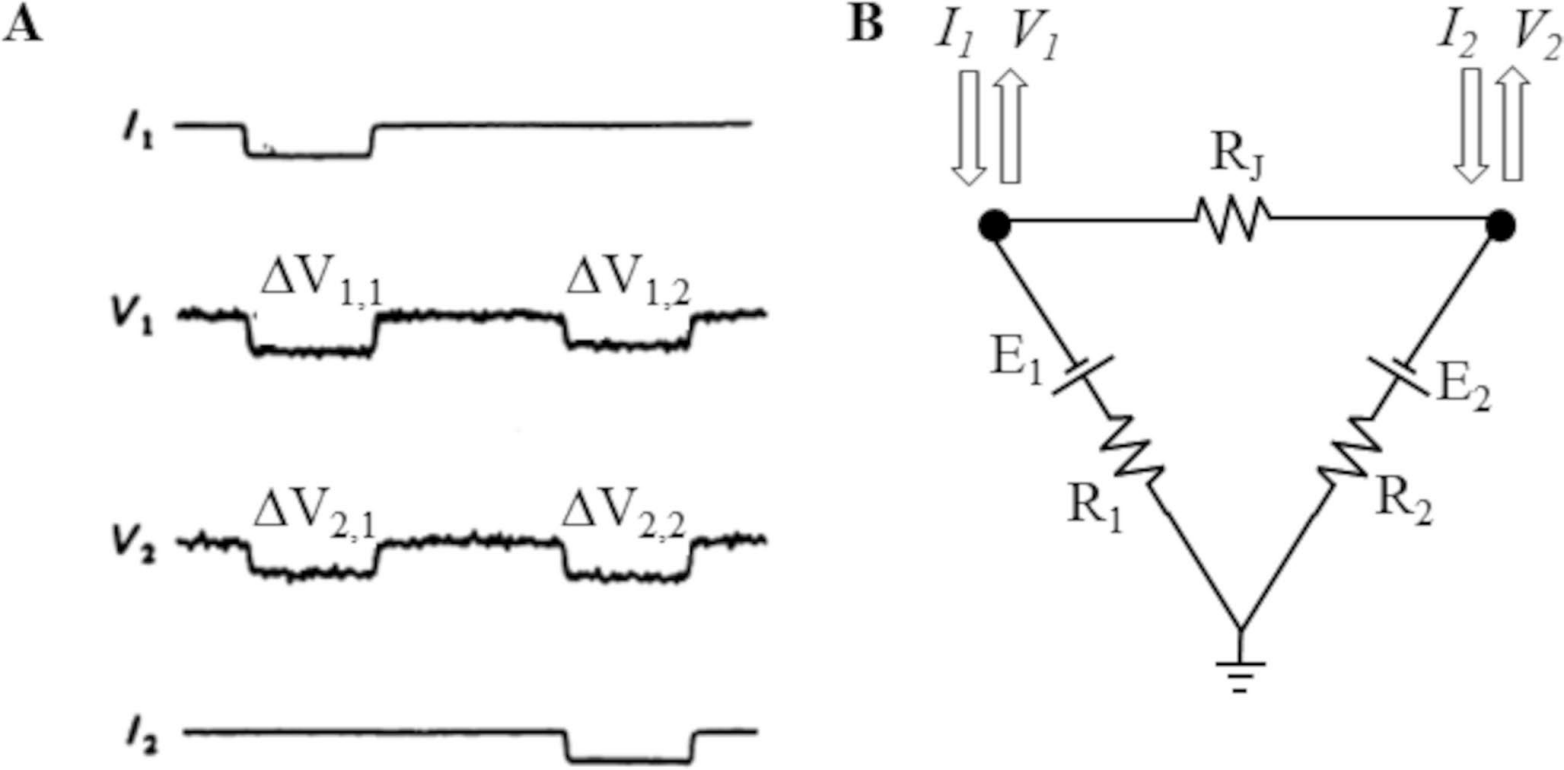
Measure of R_j_ with double current clamp. A constant current I_1_, passed through the first electrode into cell 1, produced a voltage deflection ΔV_11_ in the injected cell 1 and ΔV_21_ in the follower cell 2. Similarly, a constant current I_2_, injected into cell 2, produced a voltage deflection ΔV_22_ in the injected cell 2 and ΔV_12_ in the cell 1 (from [35]. By applying Ohm’s and Kirchhoff’s laws to these data, the values of R_1_, R_2_, and R_j_ can be derived

If a constant current I_1_ is passed through the first electrode (Fig. 4A top trace), some of the current will flow on cell_1_ and some, through R_j_, on cell_2_, leading to voltage deflections in both (ΔV_1,1_ and ΔV_2,1_). E_1_ and E_2_ can be ignored since they do not contribute to ΔVs. According to the Kirchhoff’s laws, the input resistance R_1,1_ and the coupling ratio CR_1,2_ can be derived:

3 $$\:{R}_{\mathrm{1,1}}=\:\frac{\varDelta\:{V}_{\mathrm{1,1}}}{{I}_{1}}\:=\:\frac{{R}_{1}({R}_{2}+\:{R}_{j})}{{R}_{1}+\:{R}_{2}+\:{R}_{j}}$$

4 $$\:{CR}_{\mathrm{1,2}}=\frac{\varDelta\:{V}_{\mathrm{2,1}}}{\varDelta\:{V}_{\mathrm{1,1}}}\:=\:\frac{{R}_{2}}{\:{R}_{2}+\:{R}_{j}}$$

Similarly, when a constant current I_2_ is passed through the second electrode (Fig. 4A bottom trace) and causes the voltage deflections ΔV_1,2_ and Δ V_2,2_, the input resistance R_1,2_ and the coupling ratio CR_2,1_ can be derived:

5 $$\:{R}_{\mathrm{1,2}}=\:\frac{\varDelta\:{V}_{\mathrm{2,2}}}{{I}_{2}}\:=\:\frac{{R}_{2}({R}_{1}+\:{R}_{j})}{{R}_{1}+\:{R}_{2}+\:{R}_{j}}$$

6 $$\:{CR}_{\mathrm{2,1}}=\frac{\varDelta\:{V}_{\mathrm{1,2}}}{\varDelta\:{V}_{\mathrm{2,2}}}\:=\:\frac{{R}_{1}}{\:{R}_{1}+\:{R}_{j}}$$

R_1_, R_2_, and R_J_ values can then be derived by solving the equations systems (3), (4), (5), (6) (three of the four equations are enough) [4, 35, 50, 51].

#### The measure of gap junctional electrical coupling in voltage clamp mode

The three resistances of the delta circuit can also be measured in voltage clamp mode, by clamping the membrane voltage of one cell of the pair at the resting potential, imposing voltage steps to the other cell, measuring the resulting currents and referring to the same Kirchhoff’s laws seen in current clamp mode [4, 50, 69]. Combinations of the current clamp and voltage clamp protocols can also be used to derive the same results [35].

### The electrical load

If we assume that the two coupled cells of Fig. 1 have different intrinsic (when uncoupled) resting potentials E_1_ and E_2_, then their membrane potential will be, in general:

7 $$\:{V}_{1}\:=\:{E}_{1}\:+{I}_{j}{R}_{1}$$

8 $$\:{V}_{2}\:=\:{E}_{2}\:+{I}_{j}{R}_{2}$$

And the electrotonic current I_j_ flowing across the gap junction:

9 $$\:{I}_{j}=\:\frac{\left({E}_{1}\:-\:{E}_{2}\right)}{{R}_{1}{\:+\:{R}_{2}\:+R}_{j}}$$

**Fig. 5:**
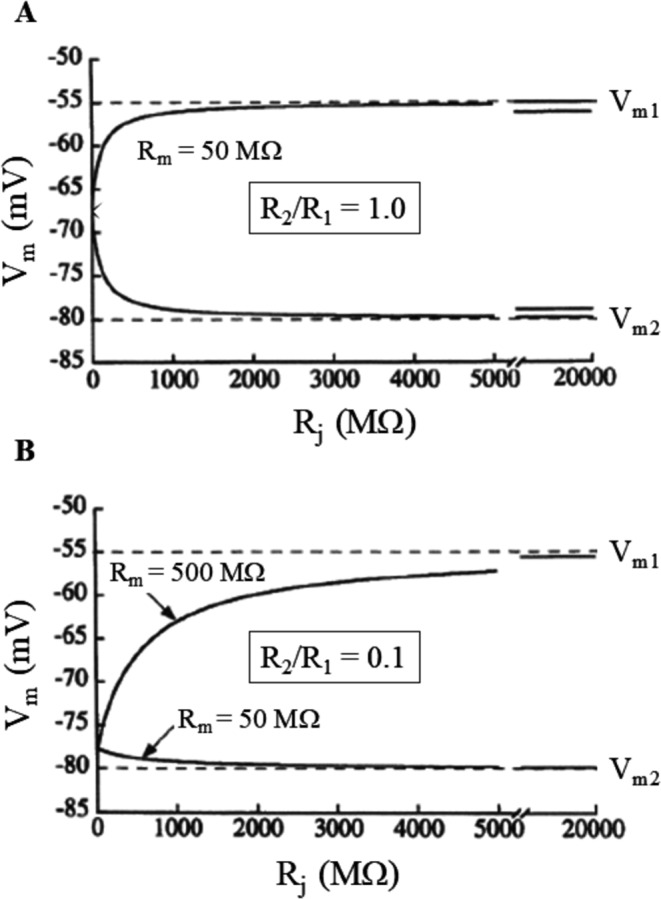
The electrical load. Mathematical model referred to the behavior of the electrical circuit of Fig. 1D (steady state). The intrinsic resting potential of cell 1 is E_1_ = −55 mV, that of cell 2 is E_2_ = −80 mV, thus V_1_ = E_1_ and V_2_ = E_2_ when cells are uncoupled (R_j_ = ∞). The steady state response of V_1_ and V_2_ as electrical coupling between cells increases (R_j_ → 0) is reported in the case when R_1_ = R_2_ (A) and when R_1_ = 10 R_2_ (B). Note that the electrical load is symmetrical (and opposite) in the case of identical membrane resistances, whereas is much higher for the cell with the greater membrane resistance in the case of different resistances (modified from Spitzer et al. 1997)

If the two cells are uncoupled (R_j_ = ∞), then I_j_ = 0 and their membrane potentials will be their intrinsic E_1_ and E_2_. As R_j_ goes from ∞ down to 0, i.e. as intercellular electrical coupling develops, then I_j_ ≠ 0 and the membrane potentials of the two cells will be given by Eqs. 7–9, where V_1_-E_1_ and V_2_-E_2_ represent the electrical loads experienced by the two. If the two cells have the same membrane resistance (R_1_ = R_2_), they will experience the same (and opposite) electrical load for any R_j_ value (Fig. 5A). If, instead, R_1_ ≠ R_2_, then the cell with higher resistance will experience the higher load (Fig. 5B).

### Source-sink relationships

**Fig. 6:**
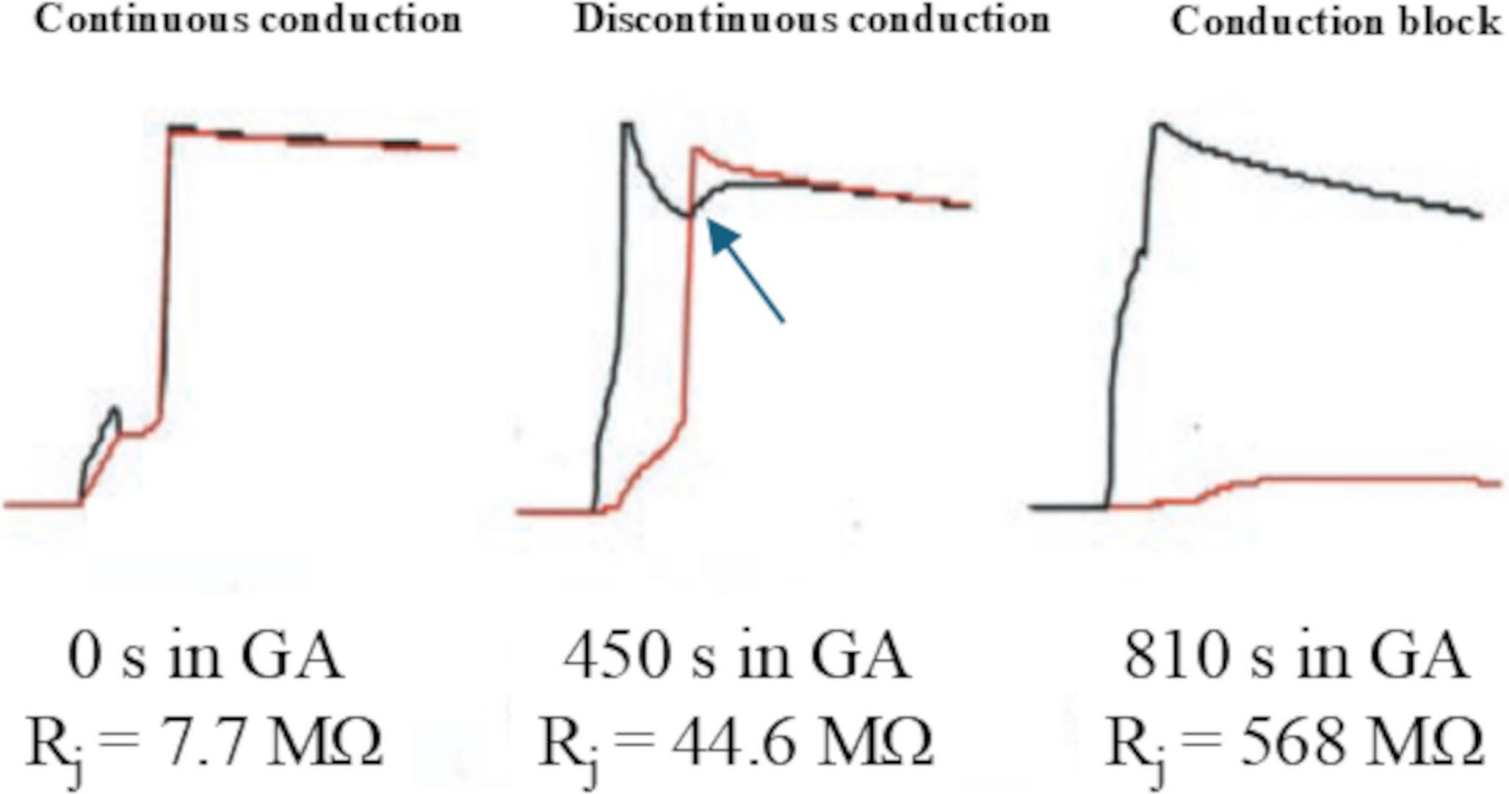
Action potential transfer in a real guinea pig ventricular cell pair. A guinea pig ventricular cell pair was perfused with 75 µM of the gap junctional uncoupler β-glycyrrhetinic acid (GA), the junctional resistance continuously measured with double-current clamp protocol described in Fig. 4, and only cell 1 paced at 1 Hz. (left) *Continuous conduction.* At start, when GA still did not have any effect (low R_j_), the threshold was reached almost simultaneously in cell 1 (black) and cell 2 (red) and AP was conducted between the two with virtually no delay. (middle) *Discontinuous conduction.* When GA caused a significant rise in R_j_, a measurable delay was found between the rising times of the two APs. (right) For longer exposure to GA, R_j_ increased further and AP conduction failed. Note the depolarizing notch in AP1 (blue arrow), due to the depolarizing source of AP2 upstroke. (modified from [96]

As already stated, the above considerations are only valid for steady state conditions, i.e. dV_m_/dt = 0, and for resting cells, i.e. for sub-threshold membrane potential changes (V_m_ < < V_th_, with V_th_ the threshold potential for excitability). More in general, the contribution of the capacitive current as well as the voltage- and time-dependence of the membrane resistance (and therefore the threshold) should also be considered, and we should refer to equation system (2) reported above. When a depolarizing stimulus current is passed in cell 1 for a given time interval (I_1_ ≠ 0, I_2_ = 0 for t_1_ < t < t_2_), this current will flow in both cells, where it will charge their membrane capacitance, more on cell 1 where current I_1_ is directly injected, less in cell 2 into whom the current is shunted through the electrotonic current I_j_. If, during the capacitive charge, V_th_ is reached in cell 1, this will fire an action potential (AP_1_) causing, into cell 2, a depolarizing electrotonic current which, depending of its strength, will depolarize V_2_ below threshold (Fig. 6, right) or reinforce its capacitive depolarization to the threshold, where AP_2_ will also be fired (Fig. 6, left and middle). For smaller R_j_ values the difference between the initial depolarization phase of the two APs, i.e. the conduction delay, will be very short or unmeasurable (Fig. 6 left), whereas, as R_j_ rises, conduction delay will increase as well (Fig. 6 middle). For further R_j_ increase not enough I_j_ current will flow to the sink cell, thus causing conduction block (Fig. 6 right).

## Results section

### The gap junctional resistance

#### R_j_in adult ventricular cell pairs

**Fig. 7:**
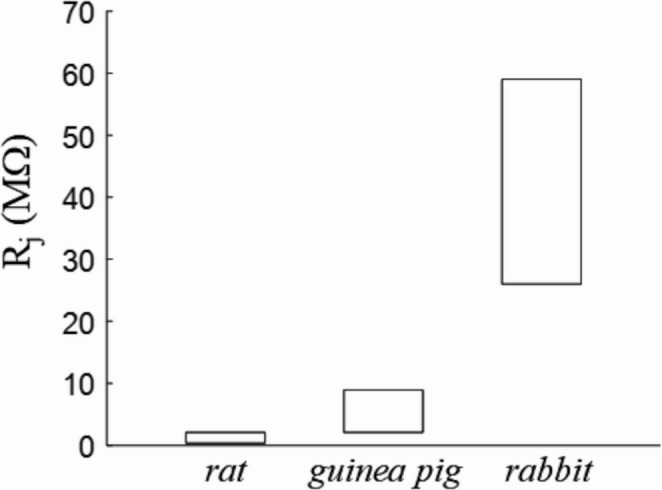
R_j_ physiological ranges. Indicative ranges covered by R_j_ in three mammalian species in control conditions. See text for description

When cells are enzymatically isolated from heart ventricles, cell pairs are found in various configurations (Fig. 2), more rarely attached end-to-end, and more frequently side-by-side, presumably due to the greater mechanical stability of this configuration [51]. The difference between the two configurations has been studied in rat hearts by Wittenberg who reported a R_j_ of 0.39 MΩ in end-to-end pairs compared to 0.83 MΩ found in side-by-side types [87]. The junctional resistance R_j_ in cell pairs has been measured by several groups, reporting values between 1.7 and 2.12 MΩ in rat [51, 80], between 2.1 MΩ and 8.9 MΩ in guinea pig [35, 49, 53, 93], and between 26 MΩ and 59 MΩ in rabbit [37, 75]. A synthetic diagram of R_j_ values in different species is reported in Fig. 7. Much higher values of R_j_ (100 MΩ) were found in rat ventricular cell pairs by White [83] possibly because they considered re-established cell pairs rather than incompletely disintegrated tissue. Also, the measured R_j_ in guinea pig cell pairs tends to increase spontaneously with time from initial values of 2–9 MΩ, up to hundreds of MΩ, presumably due to elevation of intracellular calcium concentration and/or elution of cytosolic compounds involved in connexon regulation [81].

#### Modulation of ventricular R_j_

**Fig. 8:**
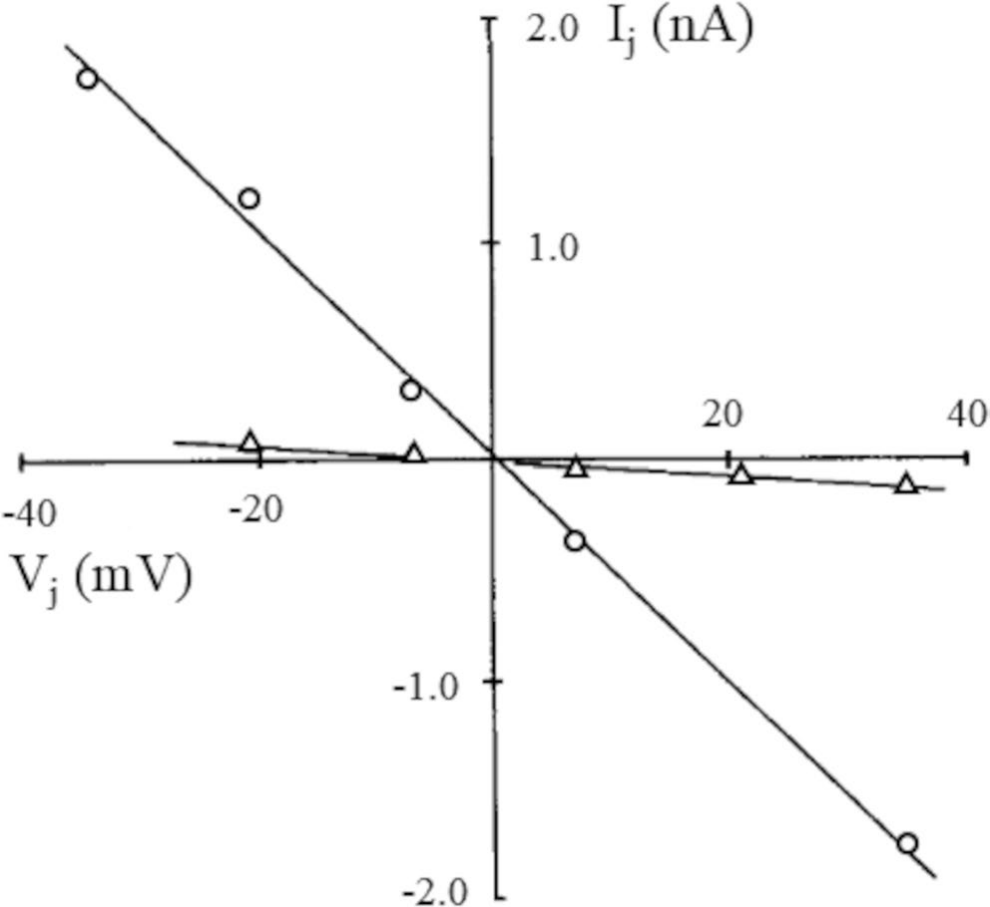
Trans-junctional current behaves ohmically in ventricular pairs. Voltage pulses of variable amplitude and polarity were applied to one cell of a pair to determine the relationship between junctional current I_j_ and trans-junctional voltage V_j_. Control (o) and after exposure to 2 µM strophantidin (Δ), a gap junctional uncoupler. From linear regression analysis the control value of R_j_ was 19 MΩ, that after strophantidin was 295 MΩ. (from [49]

All the works cited above as well as others (Rudisuli and [17, 63] reveal that R_j_ is independent from the membrane potential of the paired cells and from trans-junctional potential, as shown in double voltage clamp measurements by the ohmic nature of current-voltage relationship of the junctional membrane (Fig. 8) [49]. Also, the intercellular electrical coupling doesn’t show evidence of current rectification, i.e. junctional current flows equally well in both directions [80]. Kameyama has shown that the increase of intracellular calcium leads to the increase of R_j_ in guinea pig cell pairs [35]. The same finding has been shown by Maurer and Weingart in both rat and guinea pig cell pairs [49]. Similarly, Noma and Tsuboi have shown that both intracellular rise of calcium and magnesium cause partial cellular uncoupling in guinea pig ventricular cell pairs [57]. In the same preparation, R_j_ remained constant for extracellular pH values from 7.4 to 6.5 and increased in a dose-dependent manner for further acidification. N-alkanols, like heptanol and octanol, halothane, carbenoxolone and β-glycyrrhetinic acid have been found to reversibly uncouple cell pairs [16, 55, 73, 83, 96]. Also, the perfusion of the synthetic antiarrhythmic peptide AAP10 on cell pairs isolated from guinea pig has been found to decrease intercellular resistance either by diminishing or reversing the run-down of gap junction conductance normally observed in cell pairs of this species [53]. Desipramine has shown a similar effect, although in pairs of rabbit atrial myocytes [34]. Rabbit ventricular cells have been isolated after induction of myocardial ischemia and post-ischemic cell pairs, despite a R_j_ value not statistically different from controls (30 vs. 26 MΩ), have shown a greater proportion of poorly communicating cells [37], which can be relevant since abnormalities of junctional coupling can be implicated in cardiac arrhythmogenesis. Also, lysophosphatidylcholine (LPC), a metabolite that accumulates rapidly during cardiac ischemia, has been found to increase gap junctional resistance in guinea pig ventricular cell pairs [15]. R_j_ of ventricular cell pairs has also found to be increased (100 MΩ) in cell pairs isolated from cardiomyopathic hamsters, which, when exposed to atrial natriuretic factor, showed a further increase in R_j_ (up to 192 MΩ) [17]. Pharmacological inhibition of aerobic metabolism with the mitochondrial uncoupler 2,4-dinitrophenol increased R_j_ in guinea pig ventricular cell pairs (from 33 to 131.5 MΩ) [52]. Pharmacological treatments have been described that cause an increase in junctional electrical coupling. For example, two months administration of the angiotensin receptor blocker losartan led to enzymatic ventricular dissociations where the number of cell pairs showing very high R_j_ values (125–500 MΩ) was significantly reduced whereas the group of cell pairs with lower R_j_ values (22.2–55.5 MΩ) was significantly increased [18]. Heptanol, arachidonic acid, phorbol ester, and doxyl stearic acids induce reversible electrical uncoupling in cultured neonatal ventricular cell pairs [2, 64, 65] Munster and [7, 8, 54], while acute and chronical exposure to rotigaptide significantly increases junctional coupling [43]. Enhancement of gap junctional coupling by rotigaptide administration has been found to modulated structural and electrophysiological remodeling and reduce late arrhythmogenesis during the early healing phase in reperfused infarction [13], to suppress discordant alternans immediately preceding ventricular fibrillation [42], and favorably alter conduction patterns across Purkinje-ventricular junctions during ischemia [28], thus proving to be a promising pharmacological treatment in all these instances. Also, increase in intracellular calcium and protons causes dose dependent decrease of junctional coupling, whereas intracellular changes in magnesium and barium concentrations have no effect [22]. Cooling from 37 °C to 14 °C led to increase of R_j_ (from 20.7 to 46.7 MΩ), and from 14 °C to −2 °C to a further increase (to 57.1 MΩ) [6]. For a summary of the agents affecting gap junctional electrical coupling, see Table 1.

**Table 1:** List of agents that increase (↑) or decrease (↓) the gap junctional resistance in ventricular cell pairs

agent	type	Action on *R*_j_	Concentration
Extracellular sodium	electrolyte	↑	60 mM
Intracellular calcium	electrolyte	↑	5.0 < pCa < 7.0
Intracellular magnesium	electrolyte	↑	2.0 < pMg < 3.0
Extracellular protons	electrolyte	↑	pH < 6.5
Heptanol	7-alkanol	↑ NS	3 mM
Octanol	8-alkanol	↑ NS	0.5–1 mM
Phorbol ester	ester	↑	100–160 nM
Doxyl stearic acid	Modified form of stearic acid	↑	5–50 µM
Arachidonic acid	polyunsaturated ω−6 fatty acid	↑	100 µM
Halothane	general anaesthetic	↑ NS	3–4 mM
Carbenoxolone	glycyrrhetinic acid derivative	↑ S	50 µM
Glycyrrhetinic acid	pentacyclic triterpenoid	↑ S	40 µM
Ouabain	cardiac glycoside	↑	0.1 µM
Strophanthidin	cardiotonic steroid	↑	0.1 µM
Atrial natriuretic factor	28-amino acid peptide	↑	10 nM
Lysophosphatidyl-choline	metabolite	↑ NS	5–50 µM
2,4-dinitrophenol	mitochondrial uncoupler	↑	80 µM
AAP10	synthetic antiarrhythmic peptide	↓	10 nM
Losartan (administered)	angiotensin receptor blocker	↓	25 mg/kg/day
Cooling	temperature	↑	37 °C to −2 °C

For those agents whose action has been explicitly tested on the AP waveform without finding differences the letter S (specific) is used. Otherwise, if an action on the AP waveform has been explicitly documented, the letters NS (nonspecific) are used.

### Source-sink properties

Source-sink properties, introduced in paragraph 7, have been studied with different approaches in different preparations: real cell pairs, coupling clamped pairs, dynamic clamped pairs, and numerically simulated pairs.

#### Continuous and discontinuous conduction

When one of the cells of a ventricular cell pair is electrically stimulated to the excitation threshold, it elicits an AP (AP1) that tends to propagate to the other cell (AP2) via the depolarizing electrotonic current flowing from cell 1 (source) to cell 2 (sink) (equation system 2). Under normal coupling conditions (see R_j_ ranges in Fig. 7) no measurable delay exists between AP1 and AP2, whereas a delay develops in partially uncoupled cell pairs. In the former case we have *continuous conduction*, whereas in the latter the term *discontinuous conduction* is adopted (Fig. 6). The delay is usually measured as the difference between the time to peak of the maximum time derivative of V_m1_ and that of V_m2_. Weingart shows for example no delay in a real guinea pig ventricular cell pair with a R_j_ of 41 MΩ, and a 24 ms delay in a pair with a R_j_ of 315 MΩ [81]. The threshold is reached almost simultaneously in both cells of the pair in the case of continuous conduction (Fig. 6, left panel), whereas, in the case of discontinuous conduction, due to the higher R_j_ value, a smaller fraction of the stimulus current shunts to cell 2 which is still slowly depolarizing when cell 1 fires AP1, whose upstroke provides then extra depolarizing source to bring also cell 2 to the threshold (Fig. 6, middle panel). After AP2 is in turn fired, cell 2 becomes the depolarizing source for cell 1, which results in a characteristic depolarizing notch during AP1 repolarization(blue arrow in Fig. 6, middle panel). Finally, both APs tends to repolarize together either in continuous or discontinuous conduction. The same behavior has been shown by Zaniboni in a guinea pig cell pair where junctional uncoupling (from 7.7 to 44.6 MΩ) was induced by superfusion of β-glycyrrhetinic acid [96] (example reported in Fig. 6), and by De Groot on rabbit ventricular cell pairs using carbenoxolone [16].

#### Ion currents and calcium transient during discontinuous conduction

Among the ion currents underlying the ventricular AP, two, the L-type calcium current I_CaL_ and the transient-outward potassium current I_TO_, have been identified as major responsible in sustaining discontinuous AP conduction and, conversely, as mainly affected by the electrotonic interaction during the early phase of AP transfer.

##### L-type calcium current I_CaL_

**Fig. 9:**
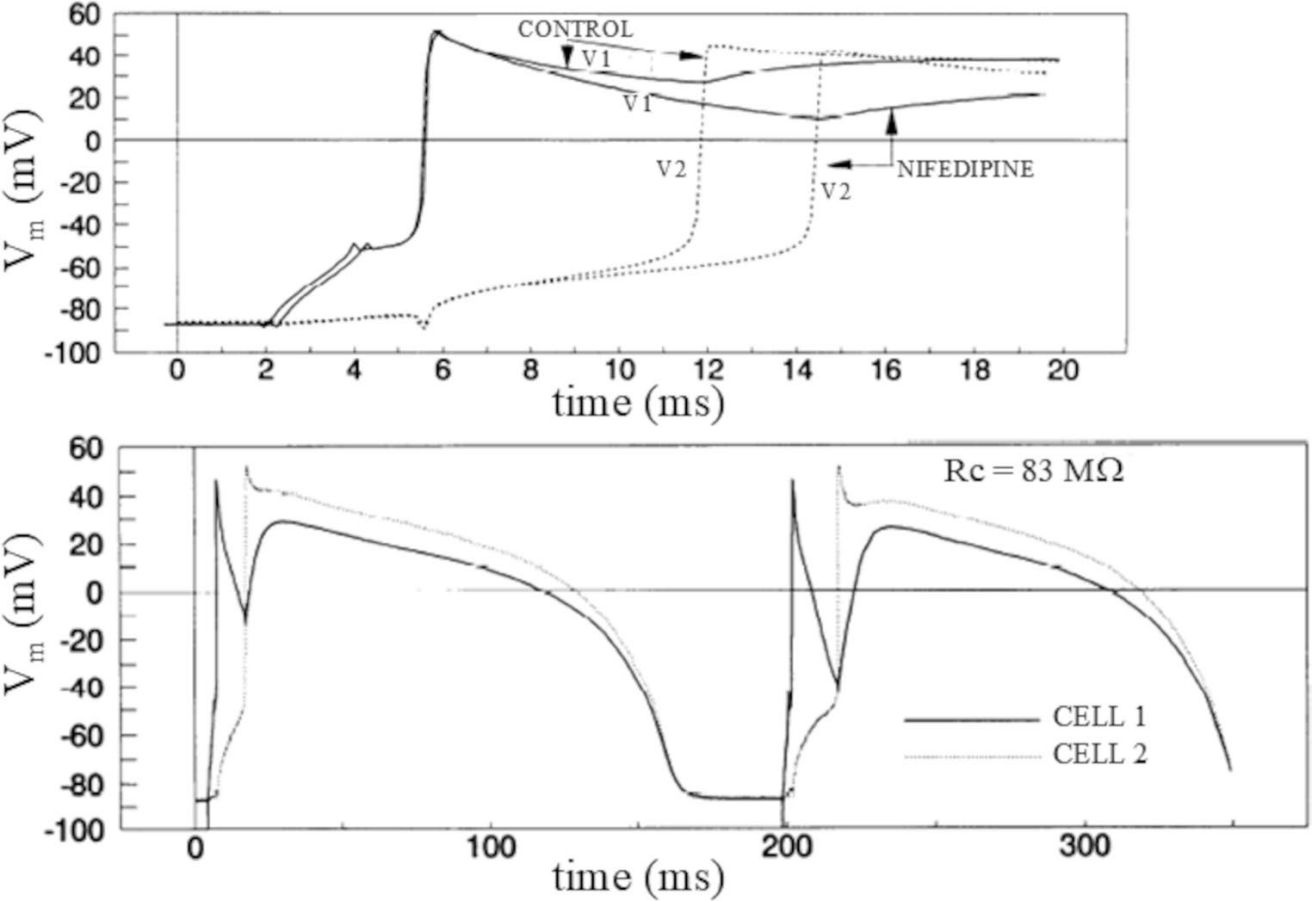
Role of calcium current in AP conduction. (Top) In a coupling clamped (Rj = 143 MΩ) pair of guinea pig ventricular myocytes, cell 1 was paced and fired an AP (solid line), which propagated to cell 2 (dotted line) with a delay of 6 ms. The perfusion of 1 µM Nifedipine caused an increase of the delay to 9 ms and an increase in the early repolarization rate of AP1. (Bottom) Similar effects were obtained during a premature stimulation (from basic cycle lenght of 500 ms to 195 ms) of cell 1 in a pair of coupling clamped (Rj = 83 MΩ) myocytes. The first on the left is the last conditioning AP at BCL = 500 ms, the second on the right is the first AP elicited prematurely. Because the incomplete removal of inactivation, I_CaL_ is reduced during the premature beat, thus causing less depolarizing junctional current, increasing the delay between the two APs, and dramatically increasing early repolarization rate in cell 1. (from [70]

In cell pairs made of two coupling clamped (Rj = 100 MΩ) guinea pig ventricular myocytes, Sugiura has measured the total membrane ionic current (I_ion_), which was negative in the paced cell, enough to elicit an AP in the same cell and provide enough negative electrotonic current to elicit an AP in the sink cell with a certain delay. Perfusion of the I_CaL_ blocker Nifedipine caused a marked reduction of I_ion_, a more rapid early repolarization in the paced cell, termed *source-loading effect* [32], and an increased delay between the two APs [70]. The same effects were obtained by reducing I_CaL_ with premature stimulations or by increasing pacing frequency (Fig. 9). Conversely, an increase of I_CaL_, by either perfusing isoproterenol or BayK 8644, promotes AP conduction by increasing the critical coupling resistance, i.e. the maximum value of R_j_ that allows AP to be conducted, as Joyner found [33] by coupling clamping a real guinea pig ventricular cell with a Luo and Rudy guinea pig ventricular AP model [45]. Thus, I_CaL_ sustains discontinuous AP conduction in a cell pair: it is legitimate to wonder what electrotonic interaction does to I_CaL_ in the same preparation. By recording the conducted AP waveforms in the leader and follower cell of a coupling clamped pair (with an R_j_ value allowing measurable conduction delay) of guinea pig ventricular myocytes and applying the corresponding waveforms in voltage clamp mode to single myocytes where I_CaL_ was blocked, Kumar and Joyner found that, during conduction delay, the L-type calcium current occurred with a larger magnitude in the leader but not in the follower cell [41]. This created an asymmetry of calcium current in the two cells, which was exacerbated by failure of AP conduction when R_j_ was increased. The larger I_CaL_ represents a metabolic load for the leader cells that needs to pump calcium out to maintain the normal intracellular ion concentration and is going to exert a significant effect on other calcium-dependent currents [66, 74] and on gap junctional resistance [83]. All these effects may play a significant role in the ability of group of cells to maintain discontinuous conduction in case of premature beats or at high pacing frequency, potentially leading to complex arrhythmogenic processes.

##### Calcium transient

Among the secondary effects expected from the increase of I_CaL_ in the leader cell of a pair during discontinuous conduction, of particular interest is the increase in the amplitude and rate of rise of the calcium transient, as found by Wagner in coupling clamped pairs made of a real guinea pig ventricular cell and the guinea pig ventricular AP model of Luo and Rudy [45, 76]. An increased calcium transient can alter the sodium-calcium exchanger current, further inactivate the calcium current and cumulatively contribute to turning off the conductance of gap junctional channels, with significance to arrhythmia formation.

##### Transient-outward potassium current I_TO_

**Fig. 10:**
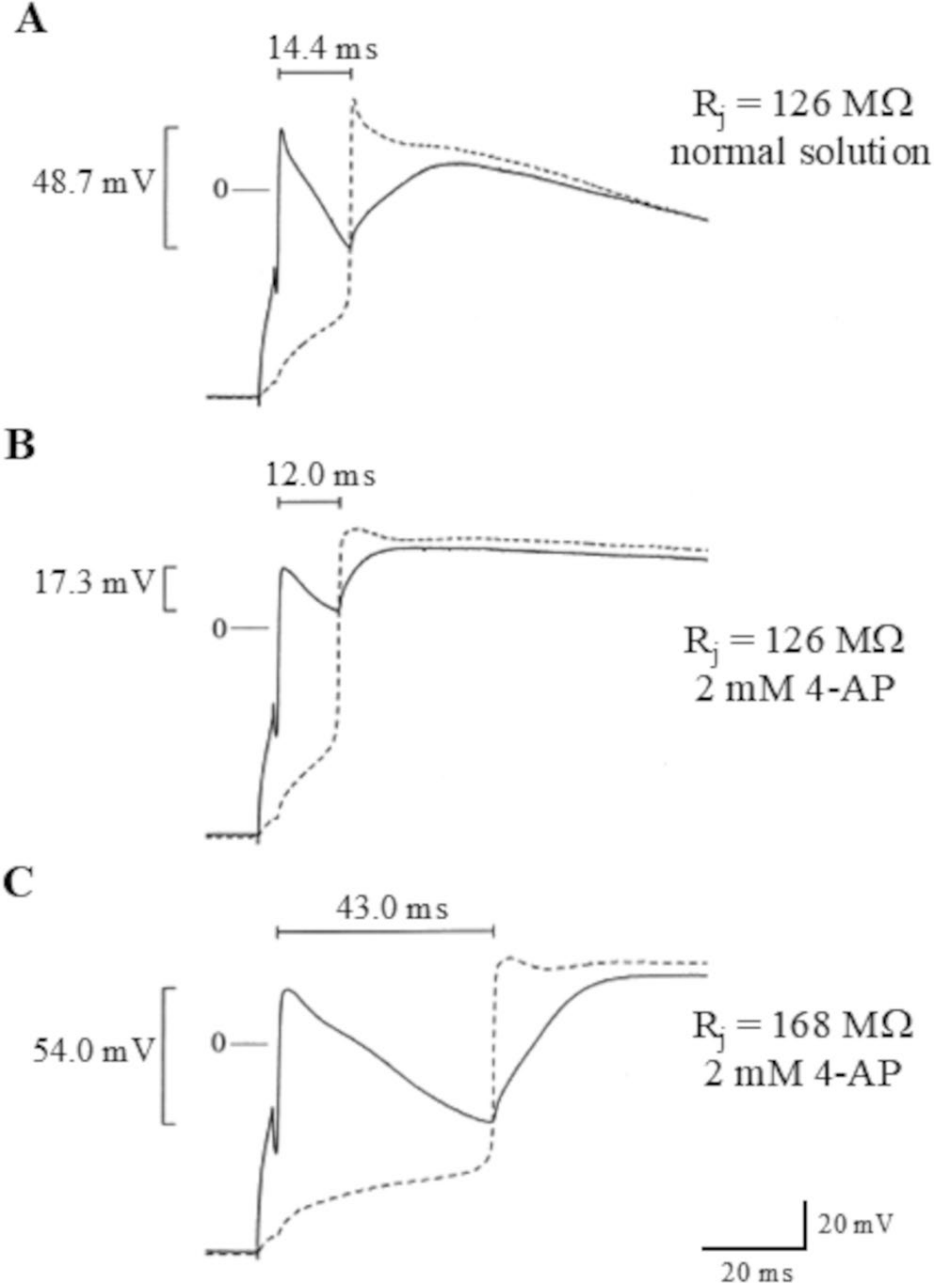
Role of transient-outward potassium current in AP conduction. (**A**) AP conduction in a pair of rabbit right ventricular myocytes coupling clamped at their critical R_j_. Leader AP is continuous line, follower dotted line. (**B**) When I_TO_ was inhibited with 4-aminopyridine and R_j_ kept at the critical value of the control condition, this led to shortening of the delay. (**C**) R_j_ was gradually increased until the new critical value in the presence of the I_TO_ blocker was found, significantly increased respect to control. (from [26]

Whereas I_CaL_ acts as a source of depolarizing current provided by the leader to the follower cell of a cell pair, the transient outward potassium current ITO acts in the opposite direction, as a source of polarizing current to the leader cell, thus opposing discontinuous AP conduction. This has been shown by Huelsing in coupling clamped pairs of rabbit right ventricular myocytes where I_TO_ was inhibited either by 4-aminopyridine superfusion, by fast pacing, or by premature stimulation [26]. I_TO_ inhibition consistently enhanced AP conduction, by decreasing conduction delay and increasing the critical R_j_, which suggests a role for this current in rate-dependent conduction abnormalities (Fig. 10). The role of I_CaL_ in favoring and of I_TO_ in contrasting discontinuous AP conduction has also been investigated by Huelsing in coupling clamped pairs made of an isolated rabbit Purkinje cell and a ventricular cell finding analogous results [23].

#### Source-sink behavior and uni-directional block

**Fig. 11:**
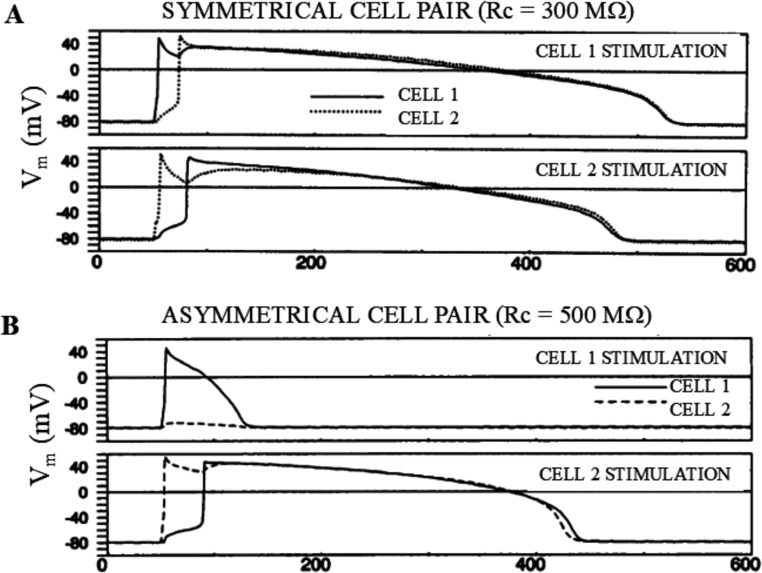
Symmetric vs. asymmetric AP conduction. (**A**) In a pair of cells with similar input resistances (22 vs. 21 MΩ), at first only cell 1 was paced (top) and, in turn, only cell 2 was paced (bottom) for the same junctional R_j_ (300 MΩ). AP conduction took place in both directions with very similar delay. (**B**) In a pair of cells with cell 1 having a much higher input resistance than cell 2 (73 vs. 30 MΩ), and for the same junctional R_j_ (500 MΩ), AP conduction failed when cell 1 was paced and succeeded when cell 2 was paced. (from [32]

An important factor involved into the electrotonic interaction between electrically connected cell pairs is the relative size of R_1_ and R_2_ in the equivalent circuit of Fig. 1. A physiological distribution of size, and therefore of intrinsic membrane resistance, is in fact documented in [23]. In the simple case when R_1_ and R_2_ values are constant and different, as explained in Fig. 5, the cell with higher R_m_ will experience, during the electrotonic coupling, the greater electrical load, i.e. the cell with lower R_m_ will tend to impose its membrane potential to the cell with higher R_m_. This appears intuitive when we consider that the cell with higher R_m_ (lower G_m_) is smaller than the one with lower R_m_ (higher G_m_), when assuming the same density of ion channels in the two. To note, we will call *input resistance* R_m_, that derived from a constant subthreshold V_m_ deflection following current injection. Lower R_m_ means, in other words, a better source of current. This fact, though in a more complex way, plays a fundamental role also when the R_m_ of each cell is the result of the complex non-linear combination of voltage- and time-dependent ion currents, and is the base of the symmetrical/asymmetrical source-sink relationship between interacting excitable cells. In fact, ventricular cells of the same size, i.e. with nearly identical input R_m_, when coupling clamped together with a given junctional R_j_, show bi-directional failure of AP conduction at very high values of R_j_, which converts to successful bidirectional conduction at lower R_j_ values. In contrast, asymmetrical coupling clamped pairs, thus with different R_m_ values, show large R_j_ ranges over which uni-directional block occurs with AP conduction successful from the larger to the smaller cell but conduction block from the smaller to the larger [32] (Fig. 11). Symmetry in input resistance means therefore similar size, current threshold, and critical junctional resistance, whereas asymmetry implies unbalance all these parameters and particularly the critical resistance, which leads in turn to R_j_ ranges allowing uni-directional block. The phenomenon of the *source-loading*, described above (paragraph 9.2), affects both symmetrical and asymmetrical pairs, though in asymmetrical pairs it affects the difference between critical resistances [32]. Analogous results on AP conduction in symmetrical and asymmetrical pairs were found by Wilders by dynamic clamping a real guinea pig ventricular cell with a Luo and Rudy guinea pig ventricular AP model [85], and by Huelsing by coupling clamping two real rabbit ventricular myocytes [23]. In the same study Huelsing studied also AP conduction in coupling clamped pairs made of a real rabbit ventricular myocyte and a real rabbit Purkinje cell of approximately the same size and found the surprising result that conduction block occurred at much lower junctional R_j_ (85 MΩ) during Purkinje-to-ventricular conduction than during ventricular-Purkinje conduction (912 MΩ). With the aid of companion numerical simulations on pairs of ventricular-Purkinje models, she showed that the difference is mainly due to the larger density of I_CaL_ and smaller density of I_TO_ in ventricular compared to Purkinje cells, which make the ventricular a better depolarizing source respect to the Purkinje cell. She also attributes the higher value of critical resistance found in ventricular-Purkinje conduction to the fact that the density of the inwardly rectifying potassium current I_K1_ is significantly lower in Purkinje cells, which causes their input resistance to be much higher and, in turn, their threshold current to be significantly lower that that of ventricular cells.

#### Electrotonic modulation of repolarization

So far, I have focused on the effects of electrical coupling on AP conduction, thus on the early phase of the AP. Electrical coupling between cell pairs has also profound implications with their entire AP waveforms, either in physiological or pathological conditions, as will be reviewed in the following paragraphs.

##### Asymmetric APD changes in ventricular pairs

**Fig. 12:**
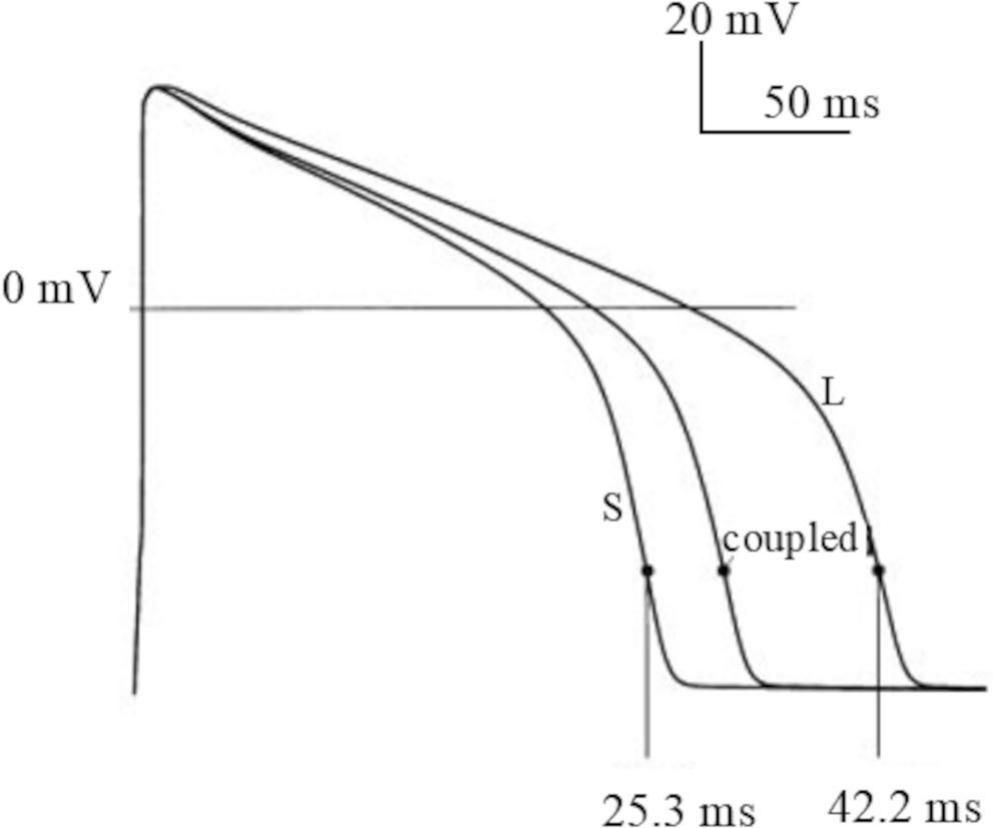
Asymmetry in APD changes. By increasing or decreasing the maximum conductance of I_kr_ in the Luo and Rudy AP model, two intrinsically short (S) and long (L) AP were obtained. When electrical coupling between the two was simulated, they reached a common coupled configuration where the shortening of the longest AP was greater than the prolongation of the shorter. (from [95]

By coupling clamping and simultaneously pacing pairs of isolated guinea pig ventricular myocytes and by companion simulations on pairs of Luo and Rudy AP models, Zaniboni has shown that, if the uncoupled cells have intrinsically longer and shorter APs, in coupled condition (e.g. Rj = 100 MΩ) they reach a common waveform, where the shortening of the longer AP is always greater than the prolongation of the shorter AP (Fig. 12) [95]. This asymmetry could not obviously be due to conduction delays since both cells were simultaneously paced, but rather to the different intrinsic time-course of membrane resistance R_m_ during the two APs. In the same study in fact, Zaniboni shows that R_m_ increases dramatically (up to 2.2 GΩ) during the AP and goes back to low diastolic values (around 9 MΩ) after AP repolarization is completed. Therefore, the loading action of the shorter AP that has already re-gained its diastolic R_m_ value accelerates the repolarization in the cell with the longer AP (and higher R_m_), forcing V_m_ trajectories into the potential range where I_K1_ terminates both APs [95]. Zaniboni has further described elsewhere the time course of R_m_ during AP [91–94, 99]. Huelsing has shown the same asymmetrical APD changes in pairs of simultaneously paced coupling clamped isolated rabbit ventricular myocytes [24].

##### Asymmetric APD changes in Purkinje-ventricular pairs

**Fig. 13:**
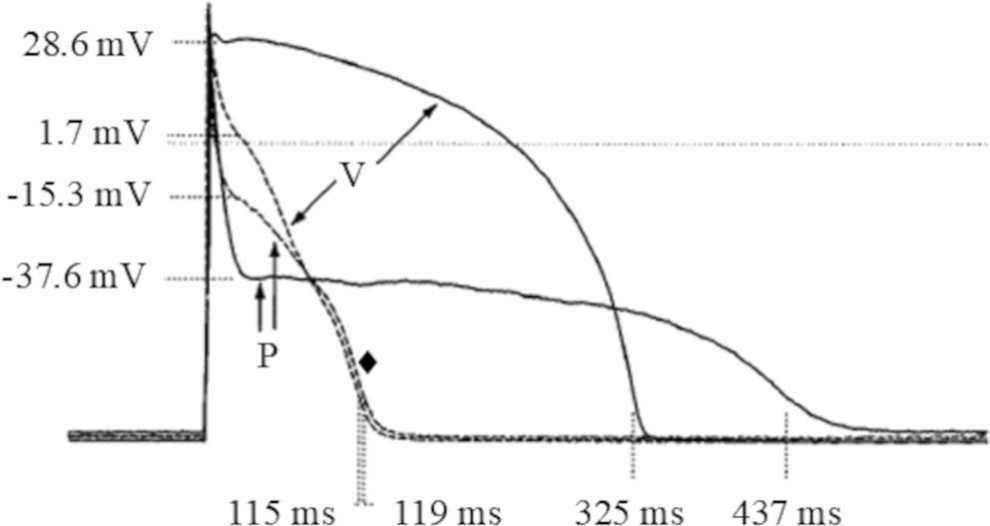
Asymmetric APD changes in ventricular-Purkinje pairs. In a pair of simultaneously paced ventricular-Purkinje myocytes (V and P) with shorter and longer intrinsic AP waveforms respectively, coupling clamping with a R_j_ = 50 MΩ led to a common coupled waveform (♦), whose APD was shorter than both intrinsic APDs. (from [24]

Huelsing have coupling clamped (R_j_ = 50 MΩ) also pairs made of ventricular and Purkinje myocytes and found a quite surprising result: in seven of eight ventricular-Purkinje pairs both APD shortened when they were coupled with respect to the uncoupled waveform (Fig. 13) [24]. Companion computer simulations have shown that the shortening of both APDs is due to the great difference in the membrane potential of the two APs during early repolarization. This is way more polarized in the Purkinje cell and provides, when coupled, a strong polarizing current flowing to the ventricular cells causing an early inactivation of L-type calcium current and early activation of inward rectifier I_K1_ which, together, lead to the dramatic shortening of the ventricular AP [24]. Thus, both the observed asymmetries in AP conduction, described in paragraph 9.3, and the APD modulation in Purkinje-ventricular pairs are mainly ascribed to heterogeneity in intrinsic early repolarization phase between the two cell types. Under ischemic conditions, the number of functional gap junctions is reduced [38], and, therefore, R_j_ is increased. Because the critical R_j_ will vary from junction to junction, this increase in R_j_ will likely induce unidirectional block at some junctions, but not at others. If conduction over the return pathway through the myocardium is slow enough to allow recovery at the sites of block, that impulse may excite the Purkinje network retrogradely, initiating circus movement reentry and arrhythmias [23].

##### Suppression of EADs and DADs

**Fig. 14:**
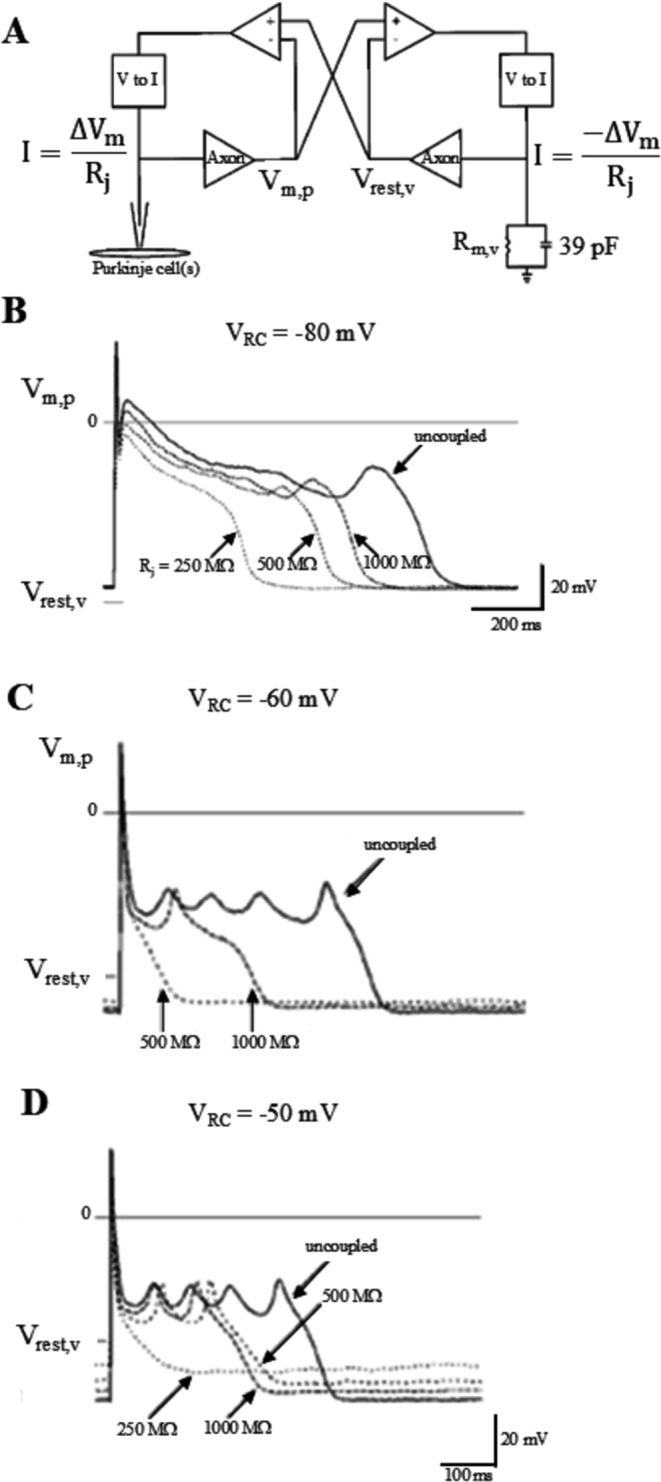
Suppression and facilitation of EADs by coupling with resting cell. (**A**) A real Purkinje cell was coupling clamped with an RC circuit with the same passive electrical properties of a ventricular myocyte (membrane potential V_RC_ of the RC circuit can be arbitrarily set). (**B** and **C**) When V_RC_ was well polarized, isoproterenol-induced EADs in the Purkinje cell were suppressed by electrotonic coupling with progressively smaller R_j_ values. (**D**) As V_RC_ became less polarized, electrical coupling did not prevent EADs formation. (from [25]

It has been proposed that early after-depolarizations (EADs) arising from Purkinje fibers can initiate triggered arrhythmias under pathological conditions [21]. By coupling clamping single Purkinje cells with an RC circuit with same passive electrical properties of a ventricular cell and variable resting potential (Fig. 14), Huelsing has found that isoproterenol-induced EADs were suppressed by coupling (R_j_ from 250 to 1000 MΩ) when the resting potential of the RC circuit was well polarized (−80 mV, Fig. 14B), but were not suppressed when the resting potential of the RC circuit was depolarized (−50 mV, Fig. 14D) [25]. This result is very important if we consider that, in the infarcted heart, the reduction of the ventricular mass due to scarring alters the ratio between Purkinje and ventricular myocytes and injury currents tend to depolarize membrane potential and alter therefore, depending on electrical coupling, the likelihood of Purkinje to developed triggered activity and arrhythmias. Suppression of EADs under coupling clamp condition has also been shown by Zaniboni between two isolated guinea pig ventricular myocytes. In uncoupled conditions one of the two cells was generating EADs due to superfusion of an I_Kr_ blocker and the other normally repolarized. By coupling clamping the two cells with a R_j_ = 100 MΩ, EADs were suppressed, and both cells repolarized along the same physiological waveform [95]. Delayed after-depolarizations (DADs) are another cause of triggered activity, whose potential in driving arrhythmias has also been studied in cell pairs. Pollard has modified parameters of the Luo and Rudy AP model to reflect conditions associated to phase 1b interval of ischemia [60]. Under these modifications suprathreshold DADs formed spontaneously after pacing. He then simulated electrical coupling between a 1b-modified ventricular myocytes with a normal one (only normal myocyte was paced) and found that, above a critical value of R_J_ (145 MΩ), DADs failed to propagate from the phase 1b myocyte to the normal one, which demonstrates the importance of source-sink relationships in triggering activity that initiate phase 1b arrhythmias in the heart.

##### Suppression of beat-to-beat APD variability

**Fig. 15:**
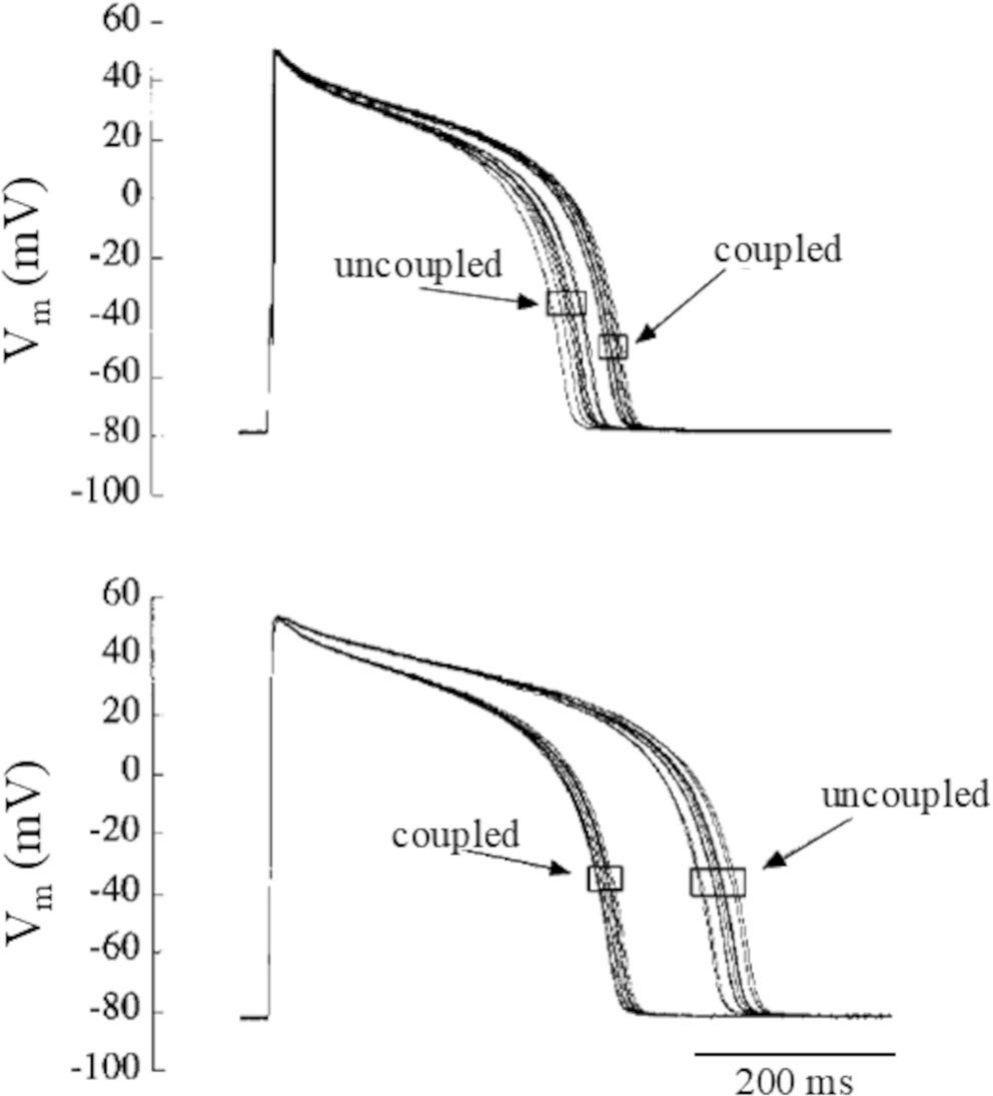
Electrotonic suppression of beat-to-beat variability of APD. Successive APs were recorded from two different isolated ventricular myocyts, one with intrinsically shorter APD (top), and the other with intrinsically longer APD (bottom), both showing intrinsic beat-to-beat APD variability. When the two cells were coupled with a R_j_ = 100 MΩ, they reached a common coupled waveform which showed a significantly smaller beat-to-beat APD variability. (from [95]

It has been shown that ventricular APs, elicited at constant frequency, have the intrinsic tendency to vary their duration on a beat-to-beat basis likely due to the stochastic behavior of ion channels [95, 98]. By coupling clamping pairs of guinea pig left ventricular myocytes, Zaniboni has shown that the electrotonic interaction significantly reduces beat-to-beat APD variability (Fig. 15) and, with that, the temporal dispersion of refractoriness, a major contribution to arrhythmogenesis [95]. In addition, Spitzer has shown that intercellular electrical coupling not only synchronize repolarization but also tends to coordinate cell shortening of the coupled myocytes, promoting synchronous contraction [68].

##### Response to high pacing frequency

**Fig. 16:**
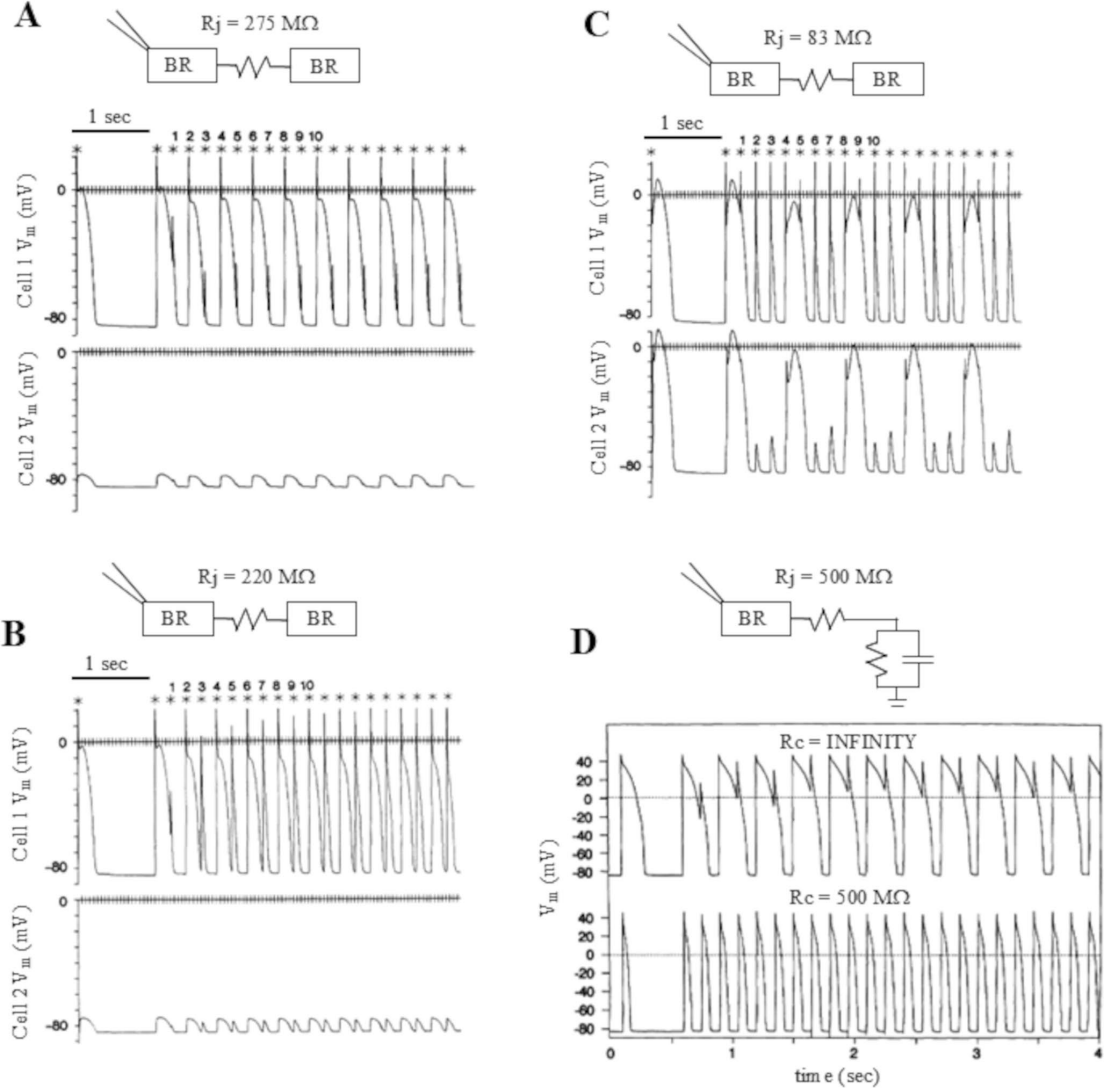
Response to high pacing frequency. (**A**-**C**) Simulated results of coupling two Beeler and Reuter models, only one paced, and (**D**) a Beeler and Reuter model with a passive RC circuit (stimulation patterns are indicated by asterisks and numbers). In all simulations the pacing CL was switched from 1000 ms to shorter values at beat 1; coupling resistance is reported in figure for each simulation. (**A**) At this level of electrical coupling, the paced cell (CL = 200 ms) showed 2:1 APD alternans, whereas the following cell only showed sub-threshold electrotonic depolarizations. (**B**) The paced cell (CL = 200 ms) showed APD 1:1 alternans, with following cell showing sub-threshold electrotonic depolarizations. (**C**) Paced cells showed 4:3 activation and following cell 4:1 activation. (**D**) Transition from CL = 500 ms to CL = 150 ms led to 1:1 APD alternans in the paced cell when uncoupled from the RC model, regular 1:1 beating when coupled. (from [71]

In a study on simulated ventricular cell pairs based on the Beeler and Reuter model [3] and on real ventricular myocytes coupling clamped with a passive RC circuit, Tan and Joyner have shown that the response of an isolated myocyte to an increased pacing frequency is strongly altered when the cell is electrically coupled to another one [71]. As the coupling resistance decreases, the paced cell becomes able to respond successfully to more rapid stimulation eliciting a full AP (Fig. 16B), though with 2:1 alternans, rather than missing every second beat (Fig. 16A). At even lower values of coupling resistance, cells exhibit arrhythmic interactions which couldn’t be predicted by the intrinsic properties of either cells of the pair (Fig. 16C). Moreover, when the electrical coupling of a single cell with a passive RC circuit was simulated, the paced cell was able, thanks to the coupling-induced dramatic electrotonic shortening of APD, to respond with 1:1 APs even with a very high value of electrical coupling (Fig. 16D). Studies on the border zone of ischemic regions have emphasized the inhomogeneous distribution of electrophysiological properties, including electrical coupling [30, 86], with small “islands” of excitable tissue variably connected to each other’s [36] or with unexcitable tissue that give rise, when measured by extracellular recordings, to irregular AP conduction and fractionated activity. This discontinuous activity can be caused by the types of interactions shown in Fig. 16 and underlie reentrant arrhythmias during post-ischemic disease.

##### Ischemia and metabolic suppression

Hallmarks of ischemia are hyperkalemia, acidosis, and hypoxia in a specific region of cardiac tissue, which typically cause spatially heterogeneous changes in local electrophysiological properties [82], thus the importance to study inter-cellular coupling in the affected regions. Ischemic conditions in ventricular cell pairs were simulated by dynamic clamping real guinea pig ventricular myocytes with the computer model of guinea pig ventricular AP of Luo and Rudy [45, 46]. The real cell, the only one paced, was exposed to a solution that included hypoxia, acidosis, and an elevated extracellular potassium concentration to mimic acute ischemia, and the critical R_j_ value that allowed AP conduction in the pair was measured [86]. The ”ischemic” solution decreased critical R_j_ value and conduction delay, whereas the same solution added with 1 µM norepinephrine increased both parameters. This suggests that release of catecholamines during ischemia in conditions of partial intercellular uncoupling can favor long conduction delays, which may allow reentrant pathways.

Inhibition of aerobic metabolism has been studied in real guinea pig ventricular cell pairs, to find the role of intercellular junctional coupling in determining failure of AP conduction in such conditions. In fact, pharmacological inhibition of aerobic metabolism with the mitochondrial uncoupler 2,4-dinitrophenol led to a decrease of membrane input resistance R_m_ followed by an increase in intercellular resistance R_j_ [52]. As R_m_ decreased, APD progressively shortened until the paced cell was unexcitable, though when AP conduction took place, the parallel increase of R_j_ was not sufficient to introduce a measurable conduction delay, thus leading to continuous conduction.

##### Ventricular-fibroblasts and ventricular-myofibroblast pairs

**Fig. 17:**
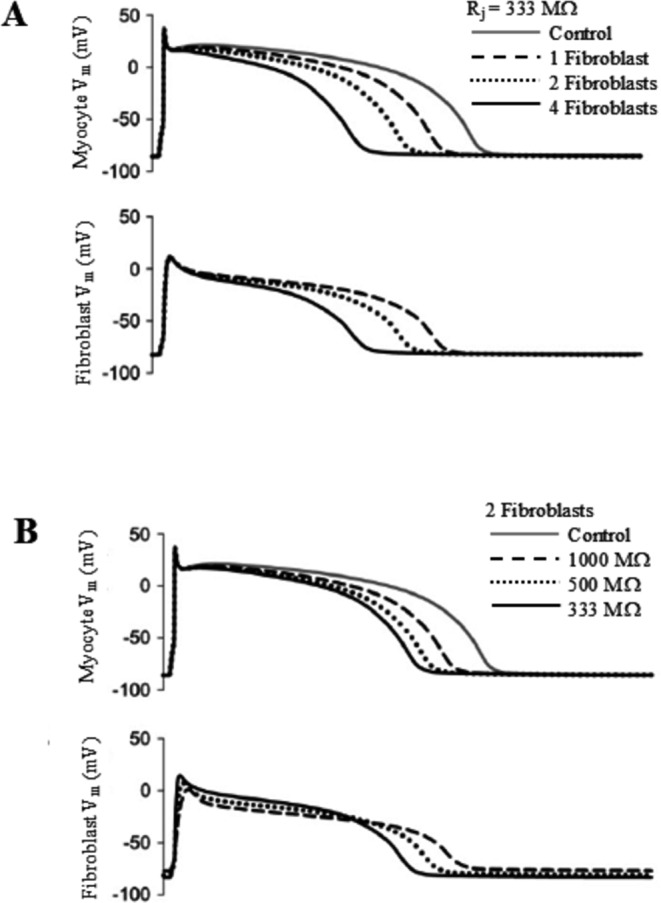
Ventricular myocyte-fibroblast coupling. (**A**) Simulated coupling of one, two, and 4 fibroblasts embedded with the four ionic currents described in the text (active fibroblst) with a ventricular myocyte, and with a junctional resistance R_j_ = 333 MΩ. (**B**) Simulated coupling of a ventricular myocyte with two active fibroblasts with junctional resistances R_j_ = 1000, 500, and 333 MΩ. (modified from Mac Cannell et al. [47]

**Fig. 18:**
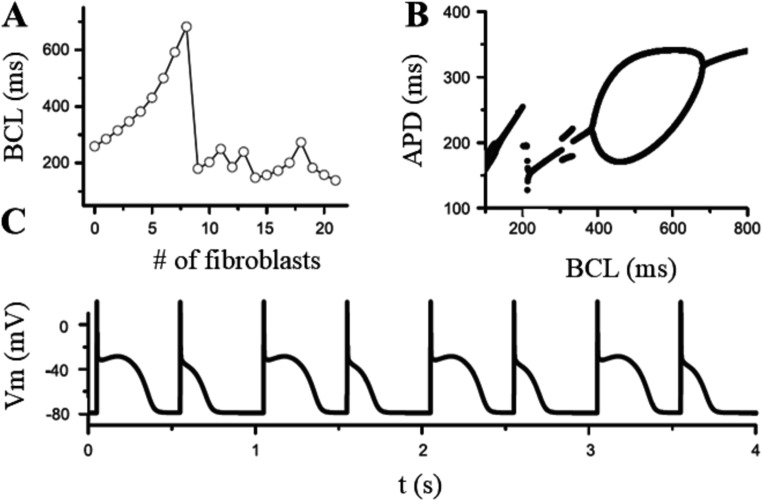
Ventricular myocyte-fibroblast coupling can promote APD alternans. The Luo and Rudy model of a myocyte was coupled with a different number of fibroblasts at different BCLs. (**A**) BCLs at which APD alternans occurred as a function of the number of fibroblasts coupled to a myocyte. (**B**) bifurcation diagram showing APD vs. BCL for a myocyte coupled to 8 fibroblasts. (**C**) AP sequence for BCL = 500 ms. (resting membrane potential of the fibroblast was − 50 mV, inter-fibroblast R_j_ = 5000 MΩ, fibroblast-myocyte R_j_ = 125 MΩ). (modified from [89, 90] B)

**Fig. 19:**
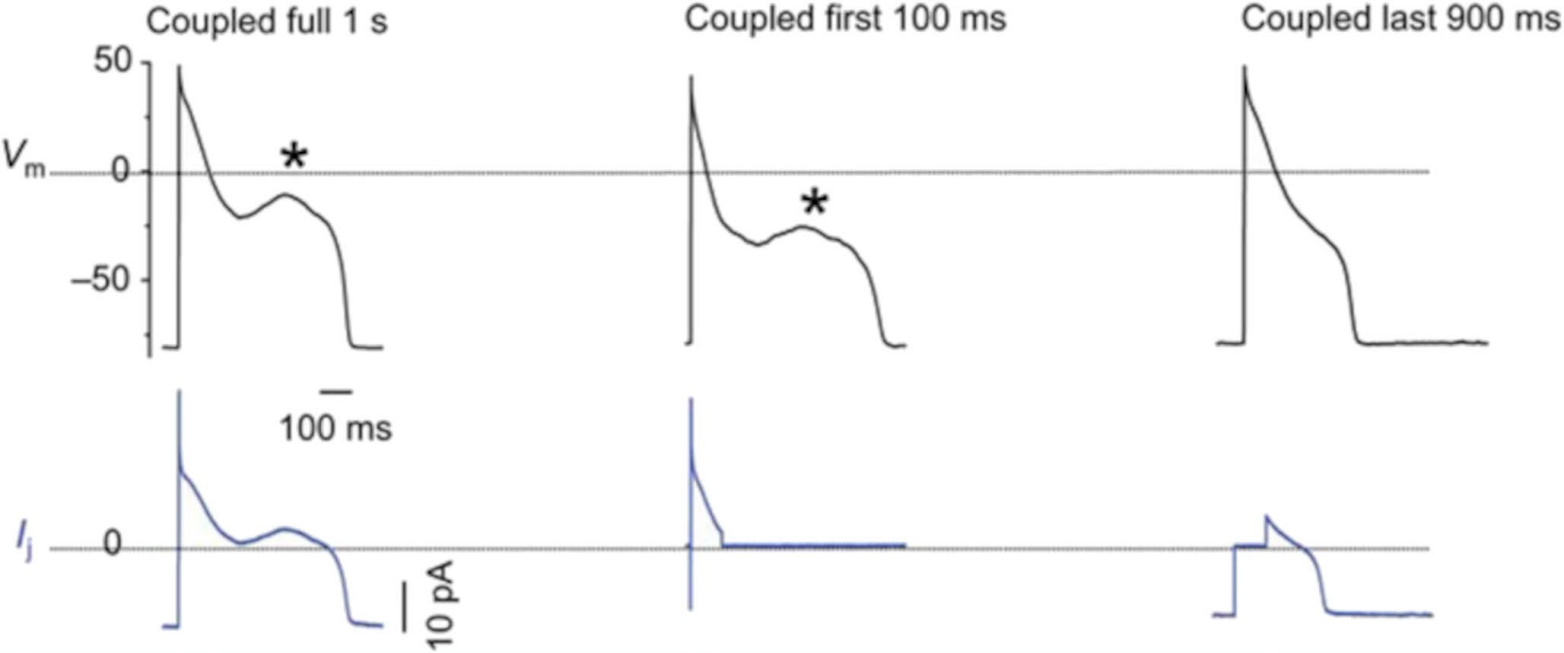
The early I_to_-like component of the junctional current promotes EADs in myocyte-myofibroblast pairs. A real patch clamped myocyte, paced at BCL = 6 s, was exposed to oxidative stress to induce EADs (star), which were suppressed at BCL = 1 s (not shown). When the myocyte was coupled to a virtual fibroblast (C_m_=6.3 pF, E_r_ = −25 mV, R_j_ = 3.3 GΩ) EADs reappears (star, left upper trace). The junctional current (left lower trace) consisted of an early transient outward I_to_-like component followed by a sustained component. When coupling was allowed only during the first 100 ms of the AP cycle, the EAD persisted (middle upper trace), whereas when coupling was allowed only during the last 900 ms of the cycle, the EAD was suppressed (right upper trace). (modified from [56]

Physiological cardiac function is controlled by complex interactions of the myocytes, extracellular matrix, and nonmyocyte cellular components, including cardiac fibroblasts, which play a key role in maintaining homeostasis in the heart [100]. Functional intercellular electrical coupling has been demonstrated among cardiac fibroblasts and between fibroblasts and ventricular myocytes [10]. The electrotonic interaction between a ten Tusscher human ventricular myocyte model [72] and one or more mammalian ventricular fibroblast models has been investigated [47]. Fibroblast were modelled either by using a simple passive RC circuit, so-called *passive fibroblast*, or by adding to the formulation (same membrane capacitance) four membrane ionic currents which have been documented in real fibroblasts [12, 67], so called *active fibroblast* 47]. The electrical coupling with the *passive fibroblast* led to prolongation of APD and no changes in the plateau, threshold for excitation or initial depolarization in the ventricular model. In contrast, coupling with the *active fibroblast* led to more pronounced effects, like reduction of the height of plateau, APD shortening, and the tendency of fibroblast potential to closely follow that of the myocyte (Fig. 17). These types of changes are likely to alter the calcium transient in the myocyte, thus modulating contraction and left-ventricular pressure development. A similar approach was adopted by Xie with different models, in turn a modified version of the Luo and Rudy 1 guinea pig model [44] and a modified version of the Mahajan rabbit model [48] for the ventricular myocyte, and a passive RC model with the same ionic currents embedded into the Mac Cannell model cited above for the fibroblast [89, 90]B). Xie analyzed the junctional current flowing from fibroblast to myocyte and identified an early pulse of transient outward current and a later background current. Depending on the relative prominence of the two components, the coupling can shorten or prolong APD, promote or suppress EADs, promote calcium-driven alternans and, by altering conduction velocity restitution, cause electromechanically concordant and discordant alternans in different regions of the cardiac tissue. Figure 18 shows, in a myocyte-fibroblast pair, the dependence of alternans from the number of fibroblasts coupled to the myocyte and from the pacing BCL. In normal adult hearts, quiescent fibroblasts outnumber myocytes, and, in response to hemo-dynamic stress or injury, differentiate into myofibroblasts that proliferate, secrete collagen, and synthesize new proteins [9]. Differentiation to myofibroblasts develops also when fibroblasts are co-cultured with cardiac myocytes. Myofibroblasts then tend to form gap junctions between each other’s and with myocytes and, since their resting potential is less negative than that of myocytes, they can depolarize cardiac tissue and induce spontaneous pacemaking [29]. Virtual fibroblasts and myofibroblasts have been dynamic clamped with real rabbit adult ventricular myocytes to test their ability to alter the electrophysiology of cardiac tissue and play an active role in arrhythmogenesis [56]. Myocytes were exposed to oxidative or ionic stress to induce bradycardia-dependent EADs. In uncoupled conditions EADs developed during slow pacing (6 s) and were suppressed by rapid pacing (1 s), whereas in the presence of electrical myofibroblast-myocyte coupling, particularly when myofibroblast resting potential was more depolarized, EADs could no longer be suppressed by rapid pacing, mainly due to the role of the early transient-outward component of the junctional current (Fig. 19, left). In the same experiment reported in figure, the time interval of junctional coupling was changed: in the first column electrical coupling lasted for all AP cycle (1 s) leading to EAD in the paced coupled myocyte. In the second column coupling lasted only the first 100 ms of AP cycle, leading again to EAD. Finally, in the third column coupling was imposed for the last 900 ms of the cycle, which led to normal repolarization, thus stressing the role of early transient outward component to the control of EAD formation.

## Limitations and future developments

The dynamics of cell pairs alone cannot explain most of the cardiac physiological or pathological events that happen on a larger space scale, like spatial heterogeneity of AP waveforms, wavefront collisions or breakthrough, spiral wave formation and meandering, reentry, or even physiological impulse propagation aspects. In the whole heart the electrotonic current is not given by the simple Ohm’s law like in the cell pair but rather by cable equations or bi-domain formalism, with the further complication of fiber orientation, intermingled fibroblasts, fat deposition, presence of vessels etc. Still, the cell pair model has given a remarkable contribution to what we now know in terms of AP propagation, unidirectional block, entrainment of repolarization, only to cite the most relevant topics. Double patch clamp recordings are the only direct way to access gap junctional electrical coupling and its regulation. Also, the cell pair provides a unique experimental model to investigate still not completely understood topics like the cellular origin of APD- and calcium transient-alternans, the source-sink dynamics in the Purkinje-ventricular junctions and in fibroblasts/myofibroblasts-ventricular interaction, and the role of ionic channels and transporters in the transition from continuous to discontinuous AP conduction. The fragmented electrograms recorded in patients with sustained ventricular tachycardia, for example, are due to discontinuous conduction that happens at the cellular level [31], and which has been described and clarified (see paragraphs 9.1 and 9.2) in ventricular cell pairs. A further example of how double patch clamp technique could profitably be used again is in the design of new pharmacological treatments, like in the case of rotigaptide. This gap junctional enhancer has been first characterized at the cell pair level [43] and only later developed in antiarrhythmic agents [13, 28, 42] that compensate for the impaired cellular coupling responsible for the genesis of cardiac arrhythmias [77].

An important limitation of double patch clamping is the fact that it requires isolating cell pairs from the yield of enzymatically dispersed cells, making the number of available pairs dependent from the action of proteolytic enzymes which is extremely difficult to control and predict. Proteolytic enzymes, in addition, can affect the degree of junctional coupling and the arrangement of coupled cell pairs, as mentioned in Chap. 8.1. These limitations, on the other hand, do not affect coupling clamp and dynamic clamp experiments, which do not require the use of real cell pairs.

When discussing AP conduction in the heart, an old but recently revisited concept should be considered, which is ephaptic conduction. It has been proposed that when gap junctional coupling is reduced, AP propagation can be supported via ephaptic coupling, a mechanism mediated by electric potential fields occurring in narrow intercellular clefts of intercalated discs between neighboring myocytes [27]. Ephaptic conduction can then enhance conduction velocity, reduce conduction block, especially when gap junctional coupling is compromised [78], and modulate the initiation and dynamics of reentrant arrhythmias [79]. In real cell pairs these clefts can easily be disrupted and are absent in coupling clamp and dynamic clamp experiments. This constitutes a further limitation of cell pairs approach, especially when considering the relevance that ephaptic conduction has gained in recent studies [1, 27, 78, 88], which rely more often in a computational approach.

Finally, if double patch clamping is less and less performed, not so ca. be said of dynamic clamping, which, by coupling real cells with mathematical models, represents, together with the numerical simulations, the frontier of cell pair experimentation in cardiac cellular electrophysiology and pharmacology [14, 58, 61]. Dynamic clamp is currently used to improve cardiomyocyte models fidelity [40], elucidate fibroblasts-myocytes interaction [5], and clarify the role of funny I_f_ current in cardiac pacemaking [62]. It is also emerging as a promising tool in the discovery of potential anti-arrhythmic targets and in pharmacological safety testing [58], in unravelling the differential roles of ion currents in regulating ventricular AP duration and arrhythmia susceptibility [19], and in many other fields of cardiac electrophysiology [14, 59, 84].

Thus, partly due to the very demanding technical expertise required to double patch clamping, partly due to its limitations and to the fast and overwhelming development of new recording techniques, particularly based on optical imaging, the double patch clamp has almost been abandoned by most of the laboratories engaged in cardiac electrophysiology research. This strengthens the need of summarizing the fundamental cellular concepts coming from cell pair approach and highlighting the future developments of this technique.

This work has benefited from the equipment and framework of the COMP-HUB Initiative, funded by the ‘Departments of Excellence’ program of the Italian Ministry for Education, University and Research (MIUR, 2018–2022).

## Supplementary Information

Below is the link to the electronic supplementary material.

Supplementary Material 1

## Data Availability

No datasets were generated or analysed during the current study.
