# Supplementary material for: The electrophysiology of ventricular cell pairs
Source: Pflugers Arch. 2025 Dec 25;478(1):15. doi: 10.1007/s00424-025-03132-1 (PMC12740972; doi:10.1007/s00424-025-03132-1)
Supplement: Supplementary file 1 — Supplementary Material 1 [file 424_2025_3132_MOESM1_ESM.docx]

**THE ELECTROPHYSIOLOGY OF VENTRICULAR CELL PAIRS**

**Massimiliano Zaniboni**

Department of Chemistry, Life Sciences and Environmental Sustainability, University of Parma (ITALY). Parco Area delle Scienze 11/A, 43100 Parma – ITALY

massimiliano.zaniboni@unipr.it

orcid.org/0000-0001-8808-2239

**KEYWORDS:** ventricular cell pairs, source-sink properties, uni-directional block, gap junctional resistance, Purkinje-ventricular junction, ventricular-fibroblast coupling

**Abstract**

Modern cardiac electrophysiology is largely focused on global electrical properties of tissue, frequently assessed by extracellular electrical recordings or optical mapping, whereas studies on the microscopic nature of action potential conduction are more rare, though fundamental to understand the whole-heart behavior. The electrophysiological dynamics of two ventricular myocytes electrically connected via gap junctions is the simplest conceivable model to study the laws governing the conduction of the electrical impulse within the heart ventricles. Since the enzymatic dispersion of cardiac cells has made it possible to isolate and investigate cardiac cell pairs directly, additional techniques have been introduced to investigate the problem, including coupling clamp, dynamic clamp, and numerical simulations. The physiology of a ventricular cell pair allows to define the basic laws governing the electrotonic interaction between cardiac cells in the absence of factors, like fiber orientation, heterogeneous spatial dispersion of electrophysiological properties, complex interaction with vessels and fibrotic tissue, that play a significant role in multicellular tissue studies. However, these laws remain at the base of our understanding of cardiac electrical functioning and should be bear in mind when dealing with the complexity of the whole organ level. This review will face both the technical issues encountered with the studies of ventricular cell pairs as well as the results found by the different authors concerning the physiological electrical coupling between cells, their physiological and pharmacological modulation, and the complex interactions between the conducted action potentials in the two cells.

**Introduction**

The cardiac tissue is largely made of excitable cells electrically connected to each other’s via gap junctions. ~~A gap junctional channel is formed by two properly docked hemichannels and each hemichannel is a hexamer of connexins. Connexin43 and Cx45 are the dominant connexins in the ventricle (Desplantez et al 2017).~~ Most of the studies on action potential (AP) propagation and on the intercellular electrical coupling have been, and still are, conducted on multicellular preparations from various regions of the heart by focusing on parameters ~~like conduction velocity and passive electrical properties,~~ ~~which are~~ related to the combination of cellular and intercellular electrical properties into the complex three-dimensional structure of the tissue, which include fiber orientation, vessels, fibroblasts and heterogeneous spatial distribution of electrophysiological properties. Techniques of enzymatic dispersion of cardiac myocytes have made it possible to access these properties at the level of isolated cell pairs. Among the yield of single cells resulting from enzymatic dispersion, cell pairs physically connected by functional gap junctions can be found (figure 1 A) and analyzed, providing the simplest possible experimental model to clarify the dynamics of electrical communication between cardiac cells. This review will focus primarily on studies concerning this model, or models that are equivalent from the electrical point of view. ~~Cell pairs have been obtained and studied from any region of cardiac tissue and,~~ Given the vastity of the literature involved, I will focus here only on ventricular cell pairs.

I will describe the different techniques used to access the electrophysiology of ventricular cell pairs, which include the double-patch clamp, the coupling-clamp, the dynamic clamp techniques, as well as the numerical simulations of cell pairs. The equivalent electrical circuit of a cell pair is essentially made by a simple triangular combination, it is also called “delta circuit” (figure 1 C and D), of three resistors, two representing the membrane resistance of the two cells of the pair, and one the electrical resistance ~~offered by~~ of the gap junctional coupling between the two. Of the three nodes of the circuit, two represent the membrane voltage of each cell and one the ground. Based on this equivalent circuit, I will describe the electrophysiological protocols~~, either in current- or in voltage-clamp mode,~~ ~~used~~ adopted to measure the three resistances, and particularly the gap junctional resistance. I will also describe the basic laws governing the relationship between the membrane voltages of the two cells when electrically connected via the gap junctions, first in the simpler case of subthreshold potentials and then during AP propagation. Results obtained with double patch clamp, coupling clamp, dynamic clamp, and computer simulations will be reviewed.

**METHODOLOGICAL SECTION**

1. **The double-patch clamp technique.** A pair of cells electrically coupled by gap junctions can be brought simultaneously in patch clamp whole cell configuration (figure 1A and B) and described by the equivalent circuit of figure 1C. Cell pairs can be found in side-by-side or in end-to-end configuration (figure 2), which are described by the same circuit. The model is based on the mutual relationship between the three resistive elements R_1_, R_2_, R_j_, described above ~~associated to the membranes of the two cells of the pair (R~~_~~1~~_ ~~and R~~_~~2~~_~~) and that associated to the gap junction (R~~_~~j~~_~~)~~. The membranes of the two cells comprehend also the corresponding electrical capacitances (C_1_ and C_2_) in parallel with their resistances. Finally, in series of each ~~membrane~~ resistance, ~~there is~~ the corresponding electromotive force (E_1_ and E_2_) is inserted, which reflects the Nernst equilibrium potential of the permeant ions and, in uncoupled conditions, represents the intrinsic resting membrane potential of the two cells. V_1_ and V_2_ are the membrane potential values read by the patch clamp amplifiers. Near resting potential conditions, i.e. for deflections of V_1_ and V_2_ of only few mV around their resting values, the main contribution to the membrane resistance is that of I_K1_ current (Carmeliet and Vereecke 2002), whose resistance is nearly constant in that voltage range, making the study of the equivalent circuit straightforward. Also, in steady state condition, i.e. when V_1_ and V_2_ do not vary in time, the capacitive component of the membrane current is zero and the equivalent circuit can be furtherly simplified to that of figure 1D. Finally, when V_1_ and/or V_2_ reach the threshold for excitation, the membrane resistive component of the equivalent circuit must be replaced by the parallel combination of all the ion currents (membrane resistances) that contribute to the AP, each one in series with the corresponding electromotive force of the reversal potential associated to the permeant ionic species. Thus, the validity of the equivalent circuit, as it is represented in figures 1 C and D, is restricted to cells at their resting potential or for membrane potential changes that do not reach the threshold for excitation.
2. **The coupling-clamp technique.** Whereas with the double-patch clamp technique the R_j_ is that, constant, of each given cell pair, an experimental approach has been developed that allows the electrical coupling of physically isolated cells via an arbitrarily chosen R_j_, so-called coupling-clamp technique (Tan and Joyner 1990). Two separated cells are brought simultaneously in patch clamp whole cell configuration and their electrodes connected via an electronic circuit that simulates the existence of a given, arbitrarily set ~~fixed~~, junctional resistance R_j_ between them (see details in figure 3). Through this experimental configuration, also described by the ~~same~~ equivalent electrical circuit of figure 1, the electrical coupling between pairs made of the same cell types or of different cell types can be studied, including cases in which one of the two cells of the pair is not excitable, like a fibroblast. Also, since, as mentioned above, resting cardiac cells behave linearly, i.e. ohmically, in the restricted voltage range (approximately ± 10 mV) around the resting potential, one of the two cells can be replaced by an electrical parallel RC circuit where an electromotive force is introduced in series to the R element to confer the desired resting membrane potential.
3. **The dynamic clamp technique.** It is an evolution of the coupling clamp technique that allows, by adopting ‘‘hard’’ real-time operating systems (Dorval et al 2001), to couple real cells to numerically reconstructed action potentials of any kind. Thus, cell pairs made, for instance, of a real rat left ventricular cell and the numerical model of the same cell type can be achieved, or cell pairs made of a real ventricular cell with the numerical model of a myofibroblast, etc.
4. **Numerically simulated cell pairs.** The numerical simulation of a single cell AP consists in solving simultaneously the differential equations of the Hodgkin-Huxley type for the membrane potential and for the gating variables (and for carriers, pumps, etc) reconstructed from voltage clamp experiments on the cell type to be described (Kogan 2009). Thus, if we omit for brevity the differential equations of the gating variables, the numerical reconstruction of a cellular AP consists in solving the following first order differential equation:

$$C_{m} \frac{d V_{m}}{dt} + \sum_{i=1}^{n} I_{ion,i} + I_{stim}=0 (1)$$

with C_m_ the electrical membrane capacitance, V_m_ the membrane potential, I_ion,i_ the ion currents, I_stim_ the stimulus current. To reconstruct the electrotonic interaction between two cells connected by a resistance R_j_, the following system must be solved, where the equations of the two single cells are coupled by the terms on the left of each equation, that simply represent the electrotonic current flowing between the two.

$$\left\{ \begin{aligned} \frac{V_{1}- V_{2}}{R_{j}} = C_{m} \frac{d V_{1}}{dt} + \sum_{i=1}^{n} I_{ion1,i} + I_{stim,1} \\ \frac{V_{2}- V_{1}}{R_{j}} = C_{m} \frac{d V_{2}}{dt} + \sum_{i=1}^{n} I_{ion2,i}+ I_{stim,2} \end{aligned} \right. (2)$$

Hodgkin-Huxley type equations like (1) or (2) are solved numerically, i.e. by discretizing time and obtaining, for each time step, the updated vector containing all the differentiated variables, by means of Euler - types methods.

1. **The measure of R_j_.**

When both cells of a physically connected cell pair are either impaled with standard microelectrodes or brought in patch clamp whole cell configuration, the electrical equivalence of figure 1C is established and R_1_, R_2_, and R_J_ can either be measured in current clamp or voltage clamp mode. As mentioned above, in steady state conditions we ~~can~~ refer to the equivalent circuit of figure 1D.

**5.1** **The measure of gap junctional electrical coupling in current clamp mode.** If a constant current I_1_ is passed through the first electrode (figure 4A top trace), some of the current will flow on cell_1_ and some, through R_j_, on cell_2_, leading to ~~a~~ voltage deflections in both (ΔV_1,1_ and ΔV_2,1_). E_1_ and E_2_ can be ignored ~~from the calculations~~ since they do not contribute to ΔVs. According to the Kirchhoff’s laws, the input resistance R_1,1_ and the coupling ratio CR_1,2_ can be derived:

$$R_{1,1}= \frac{\Delta V_{1,1}}{I_{1}} = \frac{R_{1}(R_{2}+ R_{j})}{R_{1}+ R_{2}+ R_{j}} (3)$$

$${CR}_{1,2}=\frac{\Delta V_{2,1}}{\Delta V_{1,1}} = \frac{R_{2}}{R_{2}+ R_{j}} (4)$$

Similarly, when a constant current I_2_ is passed through the second electrode (figure 4A bottom trace) and causes the voltage deflections ΔV_1,2_ and Δ V_2,2_, the input resistance R_1,2_ and the coupling ratio CR_2,1_ can be derived:

$$R_{1,2}= \frac{\Delta V_{2,2}}{I_{2}} = \frac{R_{2}(R_{1}+ R_{j})}{R_{1}+ R_{2}+ R_{j}} (5)$$

$${CR}_{2,1}=\frac{\Delta V_{1,2}}{\Delta V_{2,2}} = \frac{R_{1}}{R_{1}+ R_{j}} (6)$$

R_1_, R_2_, and R_J_ values can then be derived by solving the equations systems (3), (4), (5), (6) (three of the four equations are enough) (Bennett 1966, Kameyama 1983, Metzger and Weingart 1984, Metzger and Weingart 1985).

**5.2** **The measure of gap junctional electrical coupling in voltage clamp mode.** The three resistances of the delta circuit can also be measured in voltage clamp mode, by clamping the membrane voltage of one cell of the pair at the resting potential, imposing voltage steps to the other cell, measuring the resulting currents and referring to the same Kirchhoff’s laws seen in current clamp mode (Bennett 1966, Spray et al 1981, Metzger and Weingart 1984). Combinations of the current clamp and voltage clamp protocols can also be used to derive the same results (Kameyama 1983).

1. **The electrical load.** If we assume that the two coupled cells of figure 1 have different intrinsic (when uncoupled) resting potentials E_1_ and E_2_, then their membrane potential will be, in general:

$$V_{1} = E_{1} +I_{j}R_{1} (7)$$

$V_{2} = E_{2} +I_{j}R_{2} (8)$

And the electrotonic current I_j_ flowing across the gap junction:

$$I_{j}= \frac{\left( E_{1} - E_{2} \right)}{R_{1}{+ R_{2} +R}_{j}} (9)$$

If the two cells are uncoupled (R_j_ = ∞), then I_j_ = 0 and their membrane potentials will be their intrinsic E_1_ and E_2_. As R_j_ goes from ∞ down to 0, i.e. as ~~the~~ intercellular electrical coupling develops ~~cells couple together electrically~~, then I_j_ ≠ 0 and the membrane potentials of the two cells will be given by equations 7-9, where V_1_-E_1_ and V_2_-E_2_ represent the electrical loads experienced by the two. If the two cells have the same membrane resistance (R_1_ = R_2_), they will experience the same (and opposite) electrical load for any R_j_ value (figure 5 A). If, instead, R_1_ ≠ R_2_, then the cell with higher resistance will experience the higher load (figure 5 B).

1. **Source-sink relationships.** As already stated, the above considerations are only valid for steady state conditions, i.e. dV_m_/dt = 0, and for resting cells, i.e. for sub-threshold membrane potential changes (V_m_ << V_th_, with V_th_ the threshold potential for excitability). More in general, the contribution of the capacitive current as well as the voltage- and time-dependence of the membrane resistance (and therefore the threshold) should also be considered, and we should refer to equation system (2) reported above. ~~When one of the two cells, say cell 1, reaches the threshold for excitation before cell 2 and fires an AP, then cell 1 becomes, via I~~_~~j~~_~~, a source of depolarization for cell 2, which acts therefore as the sink for as long as V~~_~~1~~_~~>V~~_~~2~~_~~. During this time a negative (depolarizing) I~~_~~j~~_ ~~will flow to the sink from the source which, on the contrary, will experience a positive (polarizing) I~~_~~j~~_~~. More specifically~~, When a depolarizing stimulus current is passed in cell 1 for a given time interval (I_1_ ≠ 0, I_2_ = 0 for t_1_ < t < t_2_), this current will flow in both cells, where it will charge their membrane capacitance, more on cell 1 where current I_1_ is directly injected, less in cell 2 into whom the current is shunted through the electrotonic current I_j_. If, during the capacitive charge, V_th_ is reached in cell 1, this will fire an action potential (AP_1_) causing, into cell 2, a depolarizing electrotonic current which, depending of its strength, will depolarize V_2_ below threshold (figure 6, right) or ~~bust up~~ reinforce its capacitive depolarization to the threshold, where AP_2_ will also be fired (figure 6, left and middle). For smaller R_j_ values the difference between the initial depolarization phase of the two APs, i.e. the conduction delay, will be very short or unmeasurable (figure 6 left), whereas, as R_j_ rises, conduction delay will increase as well (figure 6 middle). For further R_j_ increase not enough I_j_ current will flow to the sink cell, thus causing conduction block (figure 6 right). ~~In the case reported in figure 6 the value of R~~_~~j~~_ ~~was progressively increased by perfusing the cell pair with a gap junctional uncoupling agent.~~

**RESULTS SECTION**

1. **The gap junctional resistance**
   1. **R_j_ in adult ventricular cell pairs.** When cells are enzymatically isolated from heart ventricles, cell pairs are found in various configurations (figure 2), more rarely attached end-to-end, and more frequently side-by-side, presumably due to the greater mechanical stability of this configuration (Metzger and Weingart 1985). The difference between the two configurations has been studied in rat hearts by Wittenberg who reported a R_j_ of 0.39 MΩ in end-to-end pairs compared to 0.83 MΩ found in side-by-side types (Wittenberg et al 1986). The junctional resistance R_j_ in cell pairs has been measured by several groups, reporting values between 1.7 and 2.12 MΩ in rat (Metzger and Weingart 1985, Weingart 1986), between 2.1 MΩ and 8.9 MΩ in guinea pig (Kameyama 1983, Maurer and Weingart 1987, Muller et al 1997, Zaniboni et al 2003), and between 26 MΩ and 59 MΩ in rabbit (Kieval et al 1992, Verheule 1997). A synthetic diagram of R_j_ values in different species is reported in figure 7. Much higher values of R_j_ (100 MΩ) were found in rat ventricular cell pairs by White (White et al 1985) possibly because they considered re-established cell pairs rather than incompletely disintegrated tissue. Also, ~~as the time of electrical recording goes by~~, the measured R_j_ in guinea pig cell pairs tends to increase spontaneously with time from initial values of 2-9 MΩ, up to hundreds of MΩ, presumably due to elevation of intracellular calcium concentration and/or elution of cytosolic compounds involved in connexon regulation (Weingart and Maurer 1988).
   2. **Modulation of ventricular R_j_.** All the works cited above as well as others (Rudisuli and Weingart 1989, De Mello 1998) reveal that R_j_ is independent from the membrane potential of the paired cells ~~of the pair~~ and from trans-junctional potential, as shown in double voltage clamp measurements by the ohmic nature of current-voltage relationship of the junctional membrane (figure 8) (Maurer and Weingart 1987). Also, the intercellular electrical coupling doesn’t show evidence of current rectification, i.e. junctional current flows equally well in both directions (Weingart 1986). Kameyama has shown that the increase of intracellular calcium ~~by either decreasing extracellular sodium or applying cardiac glycosides~~ leads to the increase of R_j_ in guinea pig cell pairs (Kameyama 1983). The same finding has been shown by Maurer and Weingart in both rat and guinea pig cell pairs (Maurer and Weingart 1987). Similarly, Noma and Tsuboi have shown that both intracellular rise of calcium and magnesium cause partial cellular uncoupling in guinea pig ventricular cell pairs (Noma and Tsuboi 1987). In the same preparation, R_j_ remained constant for extracellular pH values from 7.4 to 6.5 and increased in a dose-dependent manner for further acidification. N-alkanols, like heptanol and octanol, ~~and~~ halothane, carbenoxolone and β-glycyrrhetinic acid have been found to reversibly uncouple cell pairs~~, i.e. increase their R~~_~~j~~_ (Niggli et al 1989, White et al 1985, Terrar and Victory 1988, De Groot 2003, Zaniboni et al 2003). ~~Similarly, carbenoxolone and β-glycyrrhetinic acid have been shown to electrically uncouple cell pairs (De Groot 2003, Zaniboni et al 2003).~~ Also, the perfusion of the synthetic antiarrhythmic peptide AAP10 on cell pairs isolated from guinea pig has been found to decrease intercellular resistance either by diminishing or reversing the run-down of gap junction conductance normally observed in cell pairs of this species (Muller et al 1997). Desipramine has shown a similar effect, although in pairs of rabbit atrial myocytes (Jozwiak et al 2012). Rabbit ventricular cells have been isolated after induction of myocardial ischemia and post-ischemic cell pairs, despite a R_j_ value not statistically different from controls (30 vs 26 MΩ), have shown a greater proportion of poorly communicating cells (Kieval et al 1992), which can be relevant since abnormalities of junctional coupling can be implicated in cardiac arrhythmogenesis. Also, lysophosphatidylcholine (LPC), a metabolite that accumulates rapidly during cardiac ischemia, has been found to increase gap junctional resistance in guinea pig ventricular cell pairs~~, with 5 mM of extracellular magnesium exerting a protective effect against LPC-induced uncoupling~~ (Daleau 1999). R_j_ of ventricular cell pairs has also found to be increased (100 MΩ) in cell pairs isolated from cardiomyopathic hamsters, which, when exposed to atrial natriuretic factor, showed a further increase in R_j_ (up to 192 MΩ) (De Mello 1998). Pharmacological inhibition of aerobic metabolism with the mitochondrial uncoupler 2,4-dinitrophenol increased R_j_ in guinea pig ventricular cell pairs (from 33 to 131.5 MΩ) (Morley et al 1992). Pharmacological treatments have been described that cause an increase in junctional electrical coupling. For example, two months administration of the angiotensin receptor blocker losartan led to enzymatic ventricular dissociations where the number of cell pairs showing very high R_j_ values (125 – 500 MΩ) was significantly reduced whereas the group of cell pairs with lower R_j_ values (22.2 – 55.5 MΩ) was significantly increased (De Mello and Specht 2006). Heptanol, arachidonic acid, phorbol ester, and doxyl stearic acids induce reversible electrical uncoupling in cultured neonatal ventricular cell pairs (Bastide et al 1995, Schmilinsky-Fluri et al 1990, Schmilinsky-Fluri et al 1997, Munster and Weingart 1993, Burt 1989, Burt et al 1991), while acute and chronical exposure to rotigaptide significantly increases junctional coupling (Lin et al 2008). Enhancement of gap junctional coupling by rotigaptide administration has been found to modulated structural and electrophysiological remodeling and reduce late arrhythmogenesis during the early healing phase in reperfused infarction (Chowdhury et al 2021), to suppress discordant alternans immediately preceding ventricular fibrillation (Laurita et al 2024), and favorably alter conduction patterns across Purkinje-ventricular junctions during ischemia (Jabbour et al 2025), thus proving to be a promising pharmacological treatment in all these instances. Also, increase in intracellular calcium and protons causes dose dependent decrease of junctional coupling, whereas intracellular changes in magnesium and barium concentrations have no effect (Firek et al 1995). Cooling from 37°C to 14°C led to increase of R_j_ (from 20.7 to 46.7 MΩ), and from 14°C to -2°C to a further increase (to 57.1 MΩ) (Bukauskas and Weingart 1983). For a summary of the agents affecting gap junctional electrical coupling, see table 1.

| **agent** | **type** | **Action on R_j_** | **Concentration** |
| --- | --- | --- | --- |
| Extracellular sodium | electrolyte | ↑ | 60 mM |
| Intracellular calcium | electrolyte | ↑ | 5.0 < pCa < 7.0 |
| Intracellular magnesium | electrolyte | ↑ | 2.0 < pMg< 3.0 |
| Extracellular protons | electrolyte | ↑ | pH < 6.5 |
| Heptanol | 7-alkanol | ↑ NS | 3 mM |
| Octanol | 8-alkanol | ↑ NS | 0.5 - 1 mM |
| Phorbol ester | ester | ↑ | 100-160 nM |
| Doxyl stearic acid | Modified form of stearic acid | ↑ | 5 – 50 μM |
| Arachidonic acid | polyunsaturated ω-6 fatty acid | ↑ | 100 μM |
| Halothane | general anaesthetic | ↑ NS | 3 - 4 mM |
| Carbenoxolone | glycyrrhetinic acid derivative | ↑ S | 50 μM |
| Glycyrrhetinic acid | pentacyclic triterpenoid | ↑ S | 40 μM |
| Ouabain | cardiac glycoside | ↑ | 0.1 μM |
| Strophanthidin | cardiotonic steroid | ↑ | 0.1 μM |
| Atrial natriuretic factor | 28-amino acid peptide | ↑ | 10 nM |
| Lysophosphatidyl-choline | metabolite | ↑ NS | 5 – 50 μM |
| 2,4-dinitrophenol | mitochondrial uncoupler | ↑ | 80 μM |
| AAP10 | synthetic antiarrhythmic peptide | ↓ | 10 nM |
| Losartan (administered) | angiotensin receptor blocker | ↓ | 25 mg/kg/day |
| Cooling | temperature | ↑ | 37°C to -2°C |

**Table 1.** List of agents that increase (↑) or decrease (↓) the gap junctional resistance in ventricular cell pairs. For those agents whose action has been explicitly tested on the AP waveform without finding differences the letter S (specific) is used. Otherwise, if an action on the AP waveform has been explicitly documented, the letters NS (nonspecific) ~~is~~ are used.

1. **Source-sink properties**

Source-sink properties, ~~have been~~ introduced in paragraph 7, ~~and~~ have been studied with different approaches in different preparations ~~and,~~ ~~already described above~~: real cell pairs, coupling clamped pairs, dynamic clamped pairs, and numerically simulated pairs.

- 1. **Continuous and discontinuous conduction.** When one of the cells of a ventricular cell pair is electrically stimulated to the excitation threshold, it elicits an AP (AP1) that tends to propagate to the other cell (AP2) via the depolarizing electrotonic current flowing from cell 1 (source) to cell 2 (sink) (equation system 2). Under normal coupling conditions (see R_j_ ranges in figure 7) ~~there is~~ no measurable delay exists between AP1 and AP2, whereas a delay develops in partially uncoupled cell pairs. In the former case we have *continuous conduction*, whereas in the latter the term *discontinuous conduction* is adopted (figure 6). ~~I note in passing that~~ The delay is usually measured as the difference between the time to peak of the maximum time derivative of V_m1_ and that of V_m2_. Weingart shows for example no delay in a real guinea pig ventricular cell pair with a R_j_ of 41 MΩ, and a 24 ms delay in a pair with a R_j_ of 315 MΩ (Weingart and Maurer 1988). The threshold is reached almost simultaneously in both cells of the pair in the case of continuous conduction (figure 6, left panel), whereas, in the case of discontinuous conduction, due to the higher R_j_ value, a smaller fraction of the stimulus current shunts to cell 2 which is still slowly depolarizing when cell 1 fires AP1, whose upstroke provides then extra depolarizing source to bring also cell 2 to the threshold (figure 6, middle panel). After AP2 is in turn fired, cell 2 becomes the depolarizing source for cell 1, which results in a characteristic depolarizing notch during AP1 repolarization~~, leading to a spike-and-dome shape~~ (blue arrow in figure 6, middle panel). Finally, both APs tends to repolarize together either in continuous or discontinuous conduction. The same behavior has been shown by Zaniboni in a guinea pig cell pair where junctional uncoupling (from 7.7 to 44.6 MΩ) was induced by superfusion of β-glycyrrhetinic acid (Zaniboni et al 2003) (example reported in figure 6), and by De Groot on rabbit ventricular cell pairs using ~~with junctional uncoupling induced by superfusion of~~ carbenoxolone (De Groot et al 2003).
  2. **Ion currents and calcium transient during discontinuous conduction**

Among the ion currents ~~involved into~~ underlying the ventricular AP, two, the L-type calcium current I_CaL_ and the transient-outward potassium current I_TO_, have been identified as major responsible in sustaining discontinuous AP conduction and, conversely, as mainly affected by the electrotonic interaction during the early phase of AP transfer.

**L-type calcium current I_CaL_**~~. Calcium current is important in the conduction process and in the plateau of the AP, it affects the dynamics of other ion currents during the AP and is tightly modulated by the autonomic nervous system (Kumar and Joyner 1995).~~ In cell pairs made of two coupling clamped (Rj = 100 MΩ) guinea pig ventricular myocytes, Sugiura has measured the total membrane ionic current (I_ion_), which was negative in the paced cell, enough to elicit an AP in the same cell and provide enough negative electrotonic current to elicit an AP in the sink cell with a certain delay. Perfusion of the I_CaL_ blocker Nifedipine caused a marked reduction of I_ion_, a more rapid early repolarization in the paced cell, termed *source-loading effect* (Joyner et al 1991), and an increased delay between the two APs (Sugiura and Joyner 1992). The same effects were obtained by reducing I_CaL_ with premature stimulations or by increasing pacing frequency (figure 9). Conversely, an increase of I_CaL_, by either perfusing isoproterenol or BayK 8644, ~~is going to~~ promotes AP conduction by increasing the critical coupling resistance, i.e. the maximum value of R_j_ that allows AP to be conducted, as Joyner found (Joyner et al 1996) by coupling clamping a real guinea pig ventricular cell with a Luo and Rudy guinea pig ventricular AP model (Luo and Rudy 1994a/b). Thus, I_CaL_ sustains discontinuous AP conduction in a cell pair: it is legitimate to wonder what electrotonic interaction does to I_CaL_ in the same preparation. By recording the conducted AP waveforms in the leader and follower cell of a coupling clamped pair (with an R_j_ value allowing measurable conduction delay) of guinea pig ventricular myocytes and applying the corresponding waveforms in voltage clamp mode to single myocytes where I_CaL_ was blocked, Kumar and Joyner found that, during conduction delay, the L-type calcium current occurred with a larger magnitude in the leader but not in the follower cell (Kumar and Joyner 1995). ~~thus creating~~ This created an asymmetry of calcium current in the two cells, which ~~is~~ was exacerbated by failure of AP conduction when R_j_ ~~is~~ was increased. The larger I_CaL_ represents a metabolic load for the leader cells that needs to pump calcium out to maintain the normal intracellular ion concentration and is going to exert a significant effect on other calcium-dependent currents (Sheu and Fozzard 1985, Tsuboi and Kodama 1986) and on gap junctional resistance (White et al 1985). All these effects may play a significant role in the ability of group of cells to maintain discontinuous conduction in case of premature beats or at high pacing frequency, potentially leading to complex arrhythmogenic processes.

**Calcium transient.** Among the secondary effects expected from the increase of I_CaL_ in the leader cell of a pair during discontinuous conduction, of particular interest is the increase in the amplitude and rate of rise of the calcium transient, as found by Wagner in coupling clamped pairs made of a real guinea pig ventricular cell and the guinea pig ventricular AP model of Luo and Rudy (Wagner et al 2000, Luo and Rudy 1994a/b). An increased calcium transient can alter the sodium-calcium exchanger current, further inactivate the calcium current and cumulatively contribute to turning off the conductance of gap junctional channels, with significance to arrhythmia formation.

**Transient-outward potassium current I_TO_**. Whereas I_CaL_ acts as a source of depolarizing current provided by the leader to the follower cell of a cell pair, the transient outward potassium current I_TO_ acts in the opposite direction, as a source of polarizing current to the leader cell, thus opposing discontinuous AP conduction. This has been shown by Huelsing in coupling clamped pairs of rabbit right ventricular myocytes where I_TO_ was inhibited either by 4-aminopyridine superfusion, by fast pacing, or by premature stimulation (Huelsing et al 2001). I_TO_ inhibition consistently enhanced AP conduction, ~~either seen in a~~ by decreasing ~~decrease of~~ conduction delay ~~or in the~~ and increasing ~~increase in~~ the critical R_j_, which suggests ~~suggesting~~ a role for this current in rate-dependent conduction abnormalities (figure 10). The role of I_CaL_ in favoring and of I_TO_ in contrasting discontinuous AP conduction has also been investigated by Huelsing in coupling clamped pairs made of an isolated rabbit Purkinje cell and a ventricular cell finding analogous results (Huelsing et al 1998).

- 1. **Source-sink behavior and uni-directional block.**

An important factor involved into the electrotonic interaction between electrically connected cell pairs is the relative size of R_1_ and R_2_ in the equivalent circuit of figure 1. ~~Isolated cells do not have in fact the same size and~~ A physiological distribution of size, and therefore of intrinsic membrane resistance, is in fact documented in ~~the ventricle~~ ventricular enzymatic isolations (Huelsing et al 1998). In the simple case when R_1_ and R_2_ values are constant and different, as explained in figure 5, the cell with higher R_m_ will experience, during the electrotonic coupling, the greater electrical load, i.e. the cell with lower R_m_ will tend to impose its membrane potential to the cell with higher R_m_. This appears intuitive when we consider that the cell with higher R_m_ (lower G_m_) is smaller than the one with lower R_m_ (higher G_m_), when assuming the same density of ion channels in the two. To note, we will call *input resistance* R_m_, that ~~resulting~~ derived from a constant subthreshold V_m_ deflection following current injection. Lower R_m_ means, in other words, a better source of current. This fact, though in a more complex way, plays a fundamental role also when the R_m_ of each cell is the result of the complex non-linear combination of voltage- and time-dependent ion currents, and is the base of the symmetrical/asymmetrical source-sink relationship between interacting excitable cells. In fact, ventricular cells of the same size, i.e with nearly identical input R_m_, when coupling clamped together with a given junctional R_j_, show bi-directional failure of AP conduction at very high values of R_j_, which converts to successful bidirectional conduction at lower R_j_ values. In contrast, asymmetrical coupling clamped pairs, thus with different R_m_ values, ~~and therefore different size,~~ show large R_j_ ranges over which uni-directional block occurs with AP conduction successful from the larger to the smaller cell but conduction block from the smaller to the larger (Joyner et al 1991) (figure 11). Symmetry in input resistance means therefore similar size, current threshold, and critical junctional resistance, whereas asymmetry implies unbalance all these parameters and particularly the critical resistance, which leads in turn to R_j_ ranges allowing uni-directional block. The phenomenon of the *source-loading,* described above (paragraph 9.2), affects both symmetrical and asymmetrical pairs, though in asymmetrical pairs it affects the difference between critical resistances (Joyner et al 1991). Analogous results on AP conduction in symmetrical and asymmetrical pairs were found by Wilders by dynamic clamping a real guinea pig ventricular cell with a Luo and Rudy guinea pig ventricular AP model (Wilders et al 1996), and by Huelsing by coupling clamping two real rabbit ventricular myocytes (Huelsing et al 1998). In the same study Huelsing studied also AP conduction in coupling clamped pairs made of a real rabbit ventricular myocyte and a real rabbit Purkinje cell of approximately the same size ~~(evaluated in this case by comparing their electrical capacitance)~~ and found the surprising result that conduction block occurred at much lower junctional R_j_ (85 MΩ) during Purkinje-to-ventricular conduction than during ventricular-Purkinje conduction (912 MΩ). With the aid of companion numerical simulations on pairs of ventricular-Purkinje models, ~~Huelsing~~ she showed that the difference is mainly due to the larger density of I_CaL_ and smaller density of I_TO_ in ventricular compared to Purkinje cells, which make the ventricular a better depolarizing source respect to the Purkinje cell. She also attributes the higher value of critical resistance found in ventricular-Purkinje conduction to the fact that the density of the inwardly rectifying potassium current I_K1_ is significantly lower in Purkinje cells, which causes their input resistance to be much higher and, in turn, their threshold current to be significantly lower ~~(0.31 nA)~~ that that of ventricular cells ~~(0.84 nA)~~.

- 1. **Electrotonic modulation of repolarization**

So far, I have focused on the effects of electrical coupling on AP conduction, thus on the early phase of the AP. Electrical coupling between cell pairs has also profound implications with their entire AP waveforms ~~of their APs~~, either in physiological or pathological conditions, as will be reviewed in the following paragraphs.

- - 1. **Asymmetric APD changes in ventricular pairs.** By coupling clamping and simultaneously pacing pairs of isolated guinea pig ventricular myocytes and by companion simulations on pairs of Luo and Rudy AP models, Zaniboni has shown that, if the uncoupled cells have intrinsically longer and shorter APs, in coupled condition (e.g. Rj = 100 MΩ) they reach a common waveform, where the shortening of the longer AP is always greater than the prolongation of the shorter AP (figure 12) (Zaniboni et al 2000). This asymmetry could not obviously be due to conduction delays since both cells were simultaneously paced, but rather to the different intrinsic time-course of membrane resistance R_m_ during the two APs. In the same study in fact, Zaniboni shows that R_m_ increases dramatically (up to 2.2 GΩ) during the AP and goes back to low diastolic values (around 9 MΩ) after AP repolarization is completed. Therefore, the loading action of the shorter AP that has already re-gained its diastolic R_m_ value accelerates the repolarization in the cell with the longer AP (and higher R_m_), forcing V_m_ trajectories into the potential range where I_K1_ terminates both APs (Zaniboni et al 2000). Zaniboni has further described elsewhere the time course of R_m_ during AP (Zaniboni et al 2010, Zaniboni 2011, Zaniboni 2012a, Zaniboni 2012b, Zaniboni 2024). Huelsing has shown the same asymmetrical APD changes in pairs of simultaneously paced coupling clamped isolated rabbit ventricular myocytes (Huelsing et al 1999).
    2. **Asymmetric APD changes in Purkinje-ventricular pairs.** Huelsing have coupling clamped (R_j_ = 50 MΩ) also pairs made of ventricular and Purkinje myocytes and found a quite surprising result: in seven of eight ventricular-Purkinje pairs both APD shortened when they were coupled with respect to the uncoupled waveform (figure 13) (Huelsing et al 1999). Companion computer simulations have shown that the shortening of both APDs is due to the great difference in ~~the intrinsic peak plateau potentials, i.e. in~~ the membrane potential of the two ~~waveforms~~ APs during early repolarization. ~~The intrinsic peak plateau potential~~ This is way more polarized in the Purkinje cell and provides, when coupled, a strong polarizing current flowing to the ventricular cells causing an early inactivation of L-type calcium current and early activation of inward rectifier I_K1_ which, together, lead to the dramatic shortening of the ventricular AP (Huelsing et al 1999). Thus, both the observed asymmetries in AP conduction, described in paragraph 9.3, and the APD modulation in Purkinje-ventricular pairs are mainly ascribed to heterogeneity in intrinsic early repolarization phase between the two cell types. Under ischemic conditions, the number of functional gap junctions is reduced (Kleber et al 1987), and, therefore, R_j_ is increased. Because the critical R_j_ will vary from junction to junction, this increase in R_j_ will likely induce unidirectional block at some junctions, but not at others. If conduction over the return pathway through the myocardium is slow enough to allow recovery at the sites of block, that impulse may excite the Purkinje network retrogradely, initiating circus movement reentry and arrhythmias (Huelsing et al 1998).
    3. **Suppression of EADs and DADs.** It has been proposed that early after-depolarizations (EADs) arising from Purkinje fibers can initiate triggered arrhythmias under pathological conditions (El-Sherif et al 1990). By coupling clamping single Purkinje cells with an RC circuit with same passive electrical properties of a ventricular cell and variable resting potential (figure 14), Huelsing has found that isoproterenol-induced EADs~~, induced into the Purkinje cell by superfusion of 1μM isoproterenol~~, were suppressed by coupling (R_j_ from 250 to 1000 MΩ) when the resting potential of the RC circuit was well polarized (-80 mV, figure 14 B), but were not suppressed when the resting potential of the RC circuit was depolarized (-50 mV, figure 14 D) (Huelsing et al 2000). This result is very important if we consider that, in the infarcted heart, the reduction of the ventricular mass due to scarring alters the ratio between Purkinje and ventricular myocytes and injury currents tend to depolarize membrane potential and alter therefore, depending on electrical coupling, the likelihood of Purkinje to developed triggered activity and arrhythmias. Suppression of EADs under coupling clamp condition has also been shown by Zaniboni between two isolated guinea pig ventricular myocytes. In uncoupled conditions one of the two cells was generating EADs due to superfusion of an I_Kr_ blocker and the other normally repolarized. By coupling clamping the two cells with a R_j_ = 100 MΩ, EADs were suppressed, and both cells repolarized along the same physiological waveform (Zaniboni et al 2000). Delayed after-depolarizations (DADs) are another cause of triggered activity, whose potential in driving arrhythmias has also been studied in cell pairs. Pollard has modified parameters of the Luo and Rudy AP model to reflect conditions associated to phase 1b interval of ischemia (Pollard et al 2002). Under these modifications suprathreshold DADs formed spontaneously after pacing. He then simulated electrical coupling between a 1b-modified ventricular myocytes with a normal one (only normal myocyte was paced) and found that, above a critical value of R_J_ (145 MΩ), DADs failed to propagate from the phase 1b myocyte to the normal one, which demonstrates the importance of source-sink relationships in triggering activity that initiate phase 1b arrhythmias in the heart.
    4. **Suppression of beat-to-beat APD variability.** It has been shown that ventricular APs, elicited at ~~any given~~ constant frequency, have the intrinsic tendency to vary their duration on a beat-to-beat basis likely due to the stochastic behavior of ion channels (Zaniboni et al 2000, Zaniboni et al 2007). By coupling clamping pairs of guinea pig left ventricular myocytes, Zaniboni has shown that the electrotonic interaction significantly reduces beat-to-beat APD variability (figure 15) and, with that, the temporal dispersion of refractoriness, a major contribution to arrhythmogenesis (Zaniboni et al 2000). In addition, Spitzer has shown that intercellular electrical coupling not only synchronize repolarization but also tends to coordinate cell shortening of the coupled myocytes, promoting synchronous contraction (Spitzer et al 2006).
    5. **Response to high pacing frequency**. ~~The role of inhomogeneous spatial distribution of intercellular electrical coupling in generating arrhythmias has long been object of interest.~~ In a study on simulated ventricular cell pairs based on the Beeler and Reuter model (Beeler and Reuter 1977) and on real ventricular myocytes coupling clamped with a passive RC circuit, Tan and Joyner have shown that the response of an isolated myocyte to an increased pacing frequency is strongly altered when the cell is electrically coupled to another one (Tan and Joyner 1990). As the coupling resistance decreases, the paced cell becomes able to respond successfully to more rapid stimulation eliciting a full AP (figure 16 B), though with 2:1 alternans, rather than missing every second beat (figure 16 A). At even lower values of coupling resistance, cells exhibit arrhythmic interactions which couldn’t be predicted by the intrinsic properties of either cells of the pair (figure 16 C). Moreover, when the electrical coupling of a single cell with a passive RC circuit was simulated, the paced cell was able, thanks to the coupling-induced dramatic electrotonic shortening of APD, to respond with 1:1 APs even with a very high value of electrical coupling (figure 16 D). Studies on the border zone of ischemic regions have emphasized the inhomogeneous distribution of electrophysiological properties, including electrical coupling (Janse et al 1979; Wilders et al 1999), with small “islands” of excitable tissue variably connected to each other’s (Kienzle et al 1987) or with unexcitable tissue that give rise, when measured by extracellular recordings, to irregular AP conduction and fractionated activity. This discontinuous activity can be caused by the types of interactions shown in figure 16 and underlie reentrant arrhythmias during post-ischemic disease.
  1. **Ischemia and metabolic suppression.** Hallmarks of ischemia are hyperkalemia, acidosis, and hypoxia in a specific region of cardiac tissue, which typically cause spatially heterogeneous changes in local electrophysiological properties (Weiss et al 2009), thus the importance to study inter-cellular coupling in the affected regions. Ischemic conditions in ventricular cell pairs were simulated by dynamic clamping real guinea pig ventricular myocytes with the computer model of guinea pig ventricular AP of Luo and Rudy (Luo and Rudy 1994). The real cell, the only one paced, was exposed to a solution that included hypoxia, acidosis, and an elevated extracellular potassium concentration to mimic acute ischemia, and the critical R_j_ value that allowed AP conduction in the pair was measured (Wilders et al 1999). The ”ischemic” solution decreased ~~the~~ critical R_j_ value ~~from 161 down to 135 MΩ~~ and ~~also decreased~~ conduction delay ~~from 31 down to 23 ms~~, ~~Notably,~~ whereas the same solution added with 1 μM norepinephrine increased both parameters ~~critical R~~_~~j~~_ ~~from 169 to 200 MΩ, by also increasing conduction delay~~ ~~from 31 to 54 ms~~. This suggests that release of catecholamines during ischemia in conditions of partial intercellular uncoupling can favor long conduction delays, which may allow reentrant pathways.

Inhibition of aerobic metabolism has been studied in real guinea pig ventricular cell pairs, to find the role of intercellular junctional coupling in determining failure of AP conduction in such conditions. In fact, pharmacological inhibition of aerobic metabolism with the mitochondrial uncoupler 2,4-dinitrophenol led to a decrease of membrane input resistance R_m_ followed by an increase in intercellular resistance R_j_ (Morley et al 1992). As R_m_ decreased, APD progressively shortened until the paced cell was unexcitable, though when AP conduction took place, the parallel increase of R_j_ was not sufficient to introduce a measurable conduction delay, thus leading to continuous conduction.

- 1. **Ventricular-fibroblasts and ventricular-myofibroblast pairs**. Physiological cardiac function is controlled by complex interactions of the myocytes, extracellular matrix, and nonmyocyte cellular components, including cardiac fibroblasts, which play a key role

in maintaining homeostasis in the heart (Zeisberg and Kalluri 2010). Functional intercellular electrical coupling has been demonstrated among cardiac fibroblasts and between fibroblasts and ventricular myocytes (Camelliti et al 2005). The electrotonic interaction between a ten Tusscher human ventricular myocyte model (ten Tusscher et al 2006) and one or more mammalian ventricular fibroblast models has been investigated (Mac Cannell et al 2007). Fibroblast were modelled either by using a simple passive RC circuit ~~parallel combination of membrane capacitance (6.3 pF) and membrane resistance~~ ~~(10.7 GΩ)~~, so-called *passive fibroblast*, or by adding to the formulation (same membrane capacitance) four membrane ionic currents which have been documented in real fibroblasts (Chilton et al 2005, Shibukawa et al 2005), so called *active fibroblast* (Mac Cannell et al 2007). ~~The uncoupled resting membrane potential of the fibroblasts was - 49.6 mV.~~ The electrical coupling with the *passive fibroblast* led to prolongation of APD and no changes in the plateau, threshold for excitation or initial depolarization in the ventricular model. In contrast, coupling with the *active fibroblast* led to more pronounced effects, like reduction of the height of plateau, APD shortening, and the tendency of fibroblast potential to closely follow that of the myocyte (figure 17). These types of changes are likely to alter the calcium transient in the myocyte, thus modulating contraction and left-ventricular pressure development. ~~Using~~ A similar approach was adopted by Xie with different models, in turn a modified version of the Luo and Rudy 1 guinea pig model (Luo and Rudy 1991) and a modified version of the Mahajan rabbit model (Mahajan et al 2008) for the ventricular myocyte, and a passive RC model with the same ionic currents embedded into the Mac Cannell model cited above for the fibroblast (Xie et al 2009B). Xie analyzed the junctional current flowing from fibroblast to myocyte and ~~isolated~~ identified an early pulse of transient outward current and a later background current ~~(Xie et al 2009B)~~. Depending on the relative prominence of the two components, the coupling can shorten or prolong APD, promote or suppress EADs, promote calcium-driven alternans and, by altering conduction velocity restitution, ~~causing~~ cause electromechanically concordant and discordant alternans in different regions of the cardiac tissue. Figure 18 shows, in a myocyte-fibroblast pair, the dependence of alternans from the number of fibroblasts coupled to the myocyte and from the pacing BCL. In normal adult hearts, quiescent fibroblasts outnumber myocytes, and, in response to hemo-dynamic stress or injury, differentiate into myofibroblasts that proliferate, secrete collagen, and synthesize new proteins (Camelliti et al 2004). Differentiation to myofibroblasts develops also when fibroblasts are co-cultured with cardiac myocytes. Myofibroblasts then tend to form gap junctions between each other’s and with myocytes and, since their resting potential is less negative than that of myocytes, they can depolarize cardiac tissue and induce spontaneous pacemaking (Jacquemet 2006). ~~Thus,~~ Virtual fibroblasts and myofibroblasts have been dynamic clamped with real rabbit adult ventricular myocytes to test their ability to alter the electrophysiology of cardiac tissue and play an active role in arrhythmogenesis (Nguyen et al 2012). Myocytes were exposed to oxidative or ionic stress to induce bradycardia-dependent EADs. In uncoupled conditions EADs developed during slow pacing (6 s) and were suppressed by rapid pacing (1 s), whereas in the presence of electrical myofibroblast-myocyte coupling, particularly when myofibroblast resting potential was more depolarized ~~(-25 mV)~~, EADs could no longer be suppressed by rapid pacing, mainly due to the role of the early transient-outward component of the junctional current (figure 19, left). In the same experiment reported in figure, the time interval of junctional coupling was changed: in the first column electrical coupling lasted for all AP cycle (1 s) leading to EAD in the paced coupled myocyte. In the second column coupling lasted only the first 100 ms of AP cycle, leading again to EAD. Finally, in the third column coupling was imposed for the last 900 ms of the cycle, which led to normal repolarization, thus stressing the role of early transient outward component to the control of EAD formation. ~~At this regard, more~~ ~~Species-specific models of cardiomyocytes-myofibroblasts electrotonic coupling have been recently developed which take into account differences in transient outward potassium current associated with the differential Kv4.2 gene expression across species (Liu et al 2021).~~

**LIMITATIONS AND FUTURE DEVELOPMENTS**

The dynamics of cell pairs alone cannot explain most of the cardiac physiological or pathological events that happen on a larger space scale, like spatial heterogeneity of AP waveforms, wavefront collisions or breakthrough, spiral wave formation and meandering, reentry, or even physiological impulse propagation aspects. ~~All these phenomena are investigated by electrical and optical mapping, multiple microelectrodes or monophasic AP recordings, body surfaces potential mapping and other techniques.~~ In the whole heart the electrotonic current is not given ~~of course~~ by the simple Ohm’s law like in the cell pair but rather by cable equations or bi-domain formalism, with the further complication of fiber orientation, intermingled fibroblasts, fat deposition, presence of vessels etc. Still, the cell pair model~~, unfortunately in the hands of only few groups of electrophysiologists, has~~ ~~laid the foundations of what~~ has given a remarkable contribution to what we now know in terms of AP propagation, unidirectional block, entrainment of repolarization, only to cite the most relevant topics. Double patch clamp recordings are the only direct way to access gap junctional electrical coupling and its regulation ~~and have given a fundamental contribution to the knowledge in this regard~~. Also, the cell pair provides a unique experimental model to investigate still not completely understood topics like the cellular origin of APD- and calcium transient-alternans, the source-sink dynamics in the Purkinje-ventricular junctions and in fibroblasts/myofibroblasts-ventricular interaction, and the role of ionic channels and transporters in the transition from continuous to discontinuous AP conduction. The fragmented electrograms recorded in patients with sustained ventricular tachycardia, for example, are due to discontinuous conduction that happens at the cellular level (Josephson et al 1997), and which has been described and clarified (see paragraphs 9.1 and 9.2) ~~as I have shown,~~ in ventricular cell pairs. A further example of how double patch clamp technique could profitably be used again is in the design of new pharmacological treatments, like in the case of rotigaptide. This gap junctional enhancer has been first characterized at the cell pair level (Lin et al 2008) and only later developed in antiarrhythmic agents (Chowdhury et al 2021, Laurita et al 2024, Jabbour et al 2025) that compensate for the impaired cellular coupling responsible for the genesis of cardiac arrhythmias (Wang et al 2025).

An important limitation of double patch clamping is the fact that it requires isolating cell pairs from the yield of enzymatically dispersed cells, making the number of available pairs dependent from the action of proteolytic enzymes which is extremely difficult to control and predict. Proteolytic enzymes, in addition, can affect the degree of junctional coupling and the arrangement of coupled cell pairs, as mentioned in chapter 8.1. These limitations, on the other hand, ~~are less important in~~ do not affect coupling clamp and dynamic clamp experiments, which do not require the use of real cell pairs.

When discussing AP conduction in the heart, an old but ~~increasingly~~ recently revisited concept should be considered, which is ephaptic conduction. It has been proposed that when gap junctional coupling is reduced, AP propagation can be supported via ephaptic coupling, a mechanism mediated by electric potential fields occurring in narrow intercellular clefts of intercalated discs between neighboring myocytes (Ivanovic and Kucera 2021). Ephaptic conduction can then enhance conduction velocity, reduce conduction block, especially when gap junctional coupling is compromised (Wei and Mori 2025), and modulate the initiation and dynamics of reentrant arrhythmias (Wei and Tolkacheva 2025). In real cell pairs these clefts can easily be disrupted and are absent ~~at all~~ in coupling clamp and dynamic clamp experiments. This constitutes a further limitation of cell pairs approach, especially when considering the relevance that ephaptic conduction has gained in recent studies (Ivanovic and Kucera 2021, Wu et al 2025, Wei and Mori 2025, Adamas et al 2025), which rely more often in a computational approach.

Finally, if double patch clamping is less and less performed, not so ca be said of dynamic clamping, which, by coupling real cells with mathematical models, represents, together with the numerical simulations, the frontier ~~successor~~ of cell pair experimentation ~~and one of the frontiers of~~ in cardiac cellular electrophysiology and pharmacology (Clark et al 2022, Ortega et al 2018, Quach et al 2018). Dynamic clamp is currently used to improve cardiomyocyte models fidelity (Krogh-Madsen et al 2016), elucidate fibroblasts-myocytes interaction (Brown et al 2016), and clarify the role of funny I_f_ current in cardiac pacemaking (Ravagli et al 2016). It is also emerging as a promising tool in the discovery of potential anti-arrhythmic targets and in pharmacological safety testing (Ortega et al 2018), in unravelling the differential roles of ion currents in regulating ventricular AP duration and arrhythmia susceptibility (Devenyi et al 2017), and in many other fields of cardiac electrophysiology (Patel et al 2017, Clark et al 2022, Whittaker et al 2019).

Thus, partly due to the very demanding technical expertise required to double patch clamping, partly due to its limitations and to the fast and overwhelming development of new recording techniques, particularly based on optical imaging, the double patch clamp has almost been abandoned by most of the laboratories engaged in cardiac electrophysiology research. This~~, from one hand explains why the references cited in this review date relatively back in the recent past, on the other hand it~~ strengthens the need ~~to~~ of summarizing the fundamental cellular concepts coming from cell pair approach and highlighting the future developments of this technique.

This work has benefited from the equipment and framework of the COMP-HUB Initiative, funded by the ‘Departments of Excellence’ program of the Italian Ministry for Education, University and Research (MIUR, 2018-2022).

**Clinical trial number:** not applicable

**Funding:** Local Funding University of Parma, FIL 2024

**References**

1. Adams WP, Hoeker GS, Poelzing S. 2025. Flecainide sensitizes conduction to hyponatremia through an ephaptic mechanism. Heart Rhythm: S1547-5271(25)02396-3. doi: 10.1016/j.hrthm.2025.04.048.
2. Bastide B, Hervé JC, Cronier L, Délèze J. (1995). Rapid onset and calcium independence of the gap junction uncoupling induced by heptanol in cultured heart cells. Pflugers Arch. 429(3):386-93. doi: 10.1007/BF00374154.
3. Beeler GW, Reuter H. (1977). Reconstruction of the action potential of ventricular myocardial fibres J Physiol 268(1):177-210. doi: 10.1113/jphysiol.1977.sp011853.
4. Bennett MV. (1966). Physiology of electrotonic junctions. Ann N Y Acad Sci137(2):509-39. doi: 10.1111/j.1749-6632.1966.tb50178.x.
5. Brown TR, Krogh-Madsen T, Christini DJ. 2016. Illuminating Myocyte-Fibroblast Homotypic and Heterotypic Gap Junction Dynamics Using Dynamic Clamp. Biophys J; 111(4):785-797. doi: 10.1016/j.bpj.2016.06.042.
6. Burt JM. (1989). Uncoupling of cardiac cells by doxyl stearic acids specificity and mechanism of action. Am J Physiol. 256(4 Pt 1):C913-24. doi: 10.1152/ajpcell.1989.256.4.C913.
7. Burt JM, Massey KD, Minnich BN. (1991). Uncoupling of cardiac cells by fatty acids: structure-activity relationships. Comparative Study Am J Physiol. 260(3 Pt 1):C439-48. doi: 10.1152/ajpcell.1991.260.3.C439.
8. Camelliti P, Devlin GP, Matthews KG, Kohl P, Green CR. (2004). Spatially and temporally distinct expression of fibroblast connexins after sheep ventricular infarction. Cardiovasc Res 62:415–425.
9. Camelliti P, Borg TK, Kohl P. (2005). Structural and functional characterisation of cardiac fibroblasts. Cardiovasc Res 65(1):40-51. doi: 10.1016/j.cardiores.2004.08.020.
10. Carmeliet E, Vereecke J. (2002). Cardiac Cellular Electrophysiology. Kluver Academic Publisher, 1^st^ edition. p.120-122.
11. Chilton L, Ohya S, Freed D, George E, Drobic V, Shibukawa Y, MacCannell KA, Imaizumi Y, Clark RB, Dixon IMC, Giles WR. (2005). K1 currents regulate the resting membrane potential, proliferation, and contractile responses in ventricular fibroblasts and myofibroblasts. Am. J. Physiol. 288:H2931–H2939.
12. Chowdhury RA, Debney MT, Protti A, Handa BS, Patel KHK, Lyon AR, Shah AM, Ng FS, Peters NS. 2021. Rotigaptide Infusion for the First 7 Days After Myocardial Infarction-Reperfusion Reduced Late Complexity of Myocardial Architecture of the Healing Border-Zone and Arrhythmia Inducibility. J Am Heart Assoc;10(9):e020006. doi: 10.1161/JAHA.120.020006.
13. Clark AP, Wei S, Kalola D, Krogh-Madsen T, Christini DJ. 2022. An in silico-in vitro pipeline for drug cardiotoxicity screening identifies ionic pro-arrhythmia mechanisms. Br J Pharmacol. 179(20):4829-4843. doi: 10.1111/bph.15915.
14. Daleau P. (1999). Lysophosphatidylcholine, a metabolite which accumulates early in myocardium during ischemia, reduces gap junctional coupling in cardiac cells. J Mol Cell Cardiol. 31(7):1391-401. doi: 10.1006/jmcc.1999.0973.
15. De Groot JR, Veenstra T, Verkerk AO, Wilders R, Smits JPP, Wilms-Schopman FJG, Wiegerinck RF, Bourier J, Belterman CNW, Coronel R, Verheijck EE. (2003). Conduction slowing by the gap junctional uncoupler carbenoxolone. Cardiovasc Res 60(2):288-97. doi: 10.1016/j.cardiores.2003.07.004.
16. De Mello WC. (1998). Atrial natriuretic factor reduces cell coupling in the failing heart, an effect mediated by cyclic GMP. J Cardiovasc Pharmacol. 32(1):75-9. doi: 10.1097/00005344-199807000-00012.
17. De Mello WC, Specht P. (2006). Chronic blockade of angiotensin II AT1-receptors increased cell-to-cell communication, reduced fibrosis and improved impulse propagation in the failing heart. J Renin Angiotensin Aldosterone Syst. 7(4):201-5. doi: 10.3317/jraas.2006.038.
18. ~~Desplantez T. (2017). Cardiac Cx43, Cx40 and Cx45 co-assembling: involvement of connexins epitopes in formation of hemichannels and Gap junction channels. BMC Cell Biol 18(Suppl 1):3. doi: 10.1186/s12860-016-0118-4.~~
19. Devenyi RA, Ortega FA, Groenendaal W, Krogh-Madsen T, Christini DJ, Sobie EA. 2017. Differential roles of two delayed rectifier potassium currents in regulation of ventricular action potential duration and arrhythmia susceptibility. J Physiol; 595(7):2301-2317. doi: 10.1113/JP273191.
20. Do U. 2023. Adverse reactions to antiarrhythmic drugs. Cardiovasc Prev Pharmacother 2023;5(1):1-14. doi.org/10.36011/cpp.2023.5.e1
21. Dorval AD, Christini DJ, White JA. (2001). Real-Time linux dynamic clamp: a fast and flexible way to construct virtual ion channels in living cells. Ann Biomed Eng. 29(10):897-907. doi: 10.1114/1.1408929.
22. El-Sherif N, Craelius W, Boutjdir M, and Gough WB. (1990). Early afterdepolarizations and arrhythmogenesis. J Cardiovasc Electrophysiol 1: 145–160, 1990.
23. Firek L, Weingart R. (1995). Modification of gap junction conductance by divalent cations and protons in neonatal rat heart cells. J Mol Cell Cardiol. 27(8):1633-43. doi: 10.1016/s0022-2828(95)90623-1.
24. Huelsing DJ, Spitzer KW, Cordeiro JM, Pollard AE. (1998). Conduction between isolated rabbit Purkinje and ventricular myocytes coupled by a variable resistance. Am J Physiol. 274(4):H1163-73. doi: 10.1152/ajpheart.1998.274.4.H1163.
25. Huelsing DJ, Spitzer KW, Cordeiro JM, Pollard AE. (1999). Modulation of repolarization in rabbit Purkinje and ventricular myocytes coupled by a variable resistance. Am J Physiol, 276(2):H572-81. doi: 10.1152/ajpheart.1999.276.2.H572.
26. Huelsing DJ, Spitzer KW, Pollard AE. (2000). Electrotonic suppression of early afterdepolarizations in isolated rabbit Purkinje myocytes. Am J Physiol Heart Circ Physiol 279(1):H250-9. doi: 10.1152/ajpheart.2000.279.1.H250.
27. Huelsing DJ, Pollard AE, Spitzer KW. (2001). Transient outward current modulates discontinuous conduction in rabbit ventricular cell pairs. Cardiovasc Res. 49(4):779-89. doi: 10.1016/s0008-6363(00)00300-x.
28. Ivanovic E, Kucera JP. 2021. Localization of Na+ channel clusters in narrowed perinexi of gap junctions enhances cardiac impulse transmission via ephaptic coupling: a model study. J Physiol; 599(21):4779-4811. doi: 10.1113/JP282105.
29. Jabbour RJ, Behradfar E, Debney M, Nygren A, Hartley A, Efimov I, Hocini M, Peters NS, Ng FS, Vigmond EJ. 2025. Acute ischaemia and gap junction modulation modify propagation patterns across Purkinje-myocardial junctions. Front Physiol:16:1540400. doi: 10.3389/fphys.2025.1540400.
30. Jacquemet V. (2006). Pacemaker activity resulting from the coupling with nonexcitable cells. Phys Rev E Stat Nonlin Soft Matter Phys;74:011908.
31. Janse MJ, Cinca J, Moréna H, Fiolet JW, Kléber AG, de Vries GP, Becker AE, Durrer D. (1979). The "border zone" in myocardial ischemia. An electrophysiological, metabolic, and histochemical correlation in the pig heart. Circ Res 44(4):576-88. doi: 10.1161/01.res.44.4.576.
32. Josephson ME, Zimetbaum P, Huang D, Sauberman R, Monahan KM, Callans DS. 1997. Pathophysiologic substrate for sustained ventricular tachycardia in coronary artery disease. Jpn Circ J. 61(6):459-66. doi: 10.1253/jcj.61.459.
33. Joyner RW, Sugiura H, Tan RC. (1991). Unidirectional block between isolated rabbit ventricular cells coupled by a variable resistance. Biophys J. 60(5):1038-45. doi: 10.1016/S0006-3495(91)82141-5.
34. Joyner RW, Kumar R, Wilders R, Jongsma HJ, Verheijck EE, Golod DA, Van Ginneken AC, Wagner MB, Goolsby WN. (1996). Modulating L-type calcium current affects discontinuous cardiac action potential conduction. Biophys J. 71(1):237-45. doi: 10.1016/S0006-3495(96)79220-2.
35. Jozwiak J, Dietze A, Grover R, Savtschenko A, Etz C, Mohr FW, Dhein S. (2012). Desipramine prevents cardiac gap junction uncoupling. Naunyn Schmiedebergs Arch Pharmacol 385(11):1063-75. doi: 10.1007/s00210-012-0795-2.
36. Kameyama M. (1983). Electrical coupling between ventricular paired cells isolated from guinea-pig heart. J Physiol. 336:345-57. doi: 10.1113/jphysiol.1983.sp014585.
37. Kienzle MG, Tan RC, Ramza BM, Young ML, Joyner RW. (1987). Alterations in endocardial activation of the canine papillary muscle early and late after myocardial infarction. Circulation 76(4):860-74. doi: 10.1161/01.cir.76.4.860.
38. Kieval RS, Spear JF, Moore EN. (1992). Gap junctional conductance in ventricular myocyte pairs isolated from postischemic rabbit myocardium. Circ Res. 71(1):127-36. doi: 10.1161/01.res.71.1.127.
39. Kleber AG, Riegger CB, Janse MJ. (1987). Electrical uncoupling and increase of extracellular resistance after induction of ischemia in isolated, arterially perfused rabbit papillary muscle. Circ. Res. 61: 271–279.
40. Kogan BJ. (2009). Mathematical models of action potential. In: Introduction to computational cardiology. Springer 1^st^ Edition. pp. 45-80.
41. Krogh-Madsen T, Sobie EA, Christini DJ. 2016. Improving cardiomyocyte model fidelity and utility via dynamic electrophysiology protocols and optimization algorithms. J Physiol; 594(9):2525-36. doi: 10.1113/JP270618.
42. Kumar R, Joyner RW. (1995). Calcium currents of ventricular cell pairs during action potential conduction. Am J Physiol. 268(6 Pt 2):H2476-86. doi: 10.1152/ajpheart.1995.268.6.H2476.
43. Laurita KR, Piktel JS, Irish L, Nassal M, Cheng A, McCauley M, Pawlowski G, Dennis AT, Suen Y, Almahameed S, Ziv O, Gourdie RG, Wilson LD. 2024. Spontaneous Repolarization Alternans Causes VT/VF Rearrest That Is Suppressed by Preserving Gap Junctions. JACC Clin Electrophysiol;10(7 Pt 1):1271-1286. doi: 10.1016/j.jacep.2024.03.027.
44. Lin X, Zemlin C, Hennan JK, Petersen JS, Veenstra RD. (2008). Enhancement of ventricular gap-junction coupling by rotigaptide. Cardiovasc Res. 79(3):416-26. doi: 10.1093/cvr/cvn100.
45. ~~Liu F, Wu H, Yang X, Dong Y, Huang G, Genin GM, Lu TJ, Xu F. (2021). A new model of myofibroblast-cardiomyocyte interactions and their differences across species. Biophys J 120(17):3764-3775. doi: 10.1016/j.bpj.2021.06.040.~~
46. Luo CH, Rudy Y. (1991). A model of the ventricular cardiac action potential: depolarization, repolarization, and their interaction. Circ Res 68: 1501–1526.
47. Luo CH, Rudy Y. (1994). A dynamic model of the cardiac ventricular action potential. I. Simulations of ionic currents and concentration changes. Circ Res. 74(6):1071-96. doi: 10.1161/01.res.74.6.1071.
48. Luo CH, Rudy Y. (1994). A dynamic model of the cardiac ventricular action potential. II. Afterdepolarizations, triggered activity, and potentiation. Circ Res. 74(6):1097-113. doi: 10.1161/01.res.74.6.1097.
49. MacCannell KA, Bazzazi H, Chilton L, Shibukawa Y, Clark RB, Giles WR. (2007). A mathematical model of electrotonic interactions between ventricular myocytes and fibroblasts. Biophys J. 92(11):4121-32. doi: 10.1529/biophysj.106.101410.
50. Mahajan A, Shiferaw Y, Sato D, Baher A, Olcese R, Xie LH, Yang MJ, Chen PS, Restrepo JG, Karma A, Garfinkel A, Qu Z, Weiss JN. (2008). A rabbit ventricular action potential model replicating cardiac dynamics at rapid heart rates. Biophys J 94: 392–410.
51. Maurer P, Weingart R. (1987). Cell pairs isolated from adult guinea pig and rat hearts: effects of [Ca2+]i on nexal membrane resistance. Pflugers Arch. 409(4-5):394-402. doi: 10.1007/BF00583793.
52. Metzger P, Weingart R. (1984). Electric current flow in a two-cell preparation from Chironomus salivary glands. J Physiol, 346:599-619. doi: 10.1113/jphysiol.1984.sp015044.
53. Metzger P, Weingart R. (1985). Electric current flow in cell pairs isolated from adult rat hearts. J Physiol, 366:177-95. doi: 10.1113/jphysiol.1985.sp015791.
54. Morley GE, Anumonwo JM, Delmar M. (1992). Effects of 2,4-dinitrophenol or low [ATP]i on cell excitability and action potential propagation in guinea pig ventricular myocytes. Circ Res, 71(4):821-30. doi: 10.1161/01.res.71.4.821.
55. Müller A, Gottwald M, Tudyka T, Linke W, Klaus W, Dhein S. (1997). Increase in gap junction conductance by an antiarrhythmic peptide. Eur J Pharmacol 327(1):65-72. doi: 10.1016/s0014-2999(97)89679-3.
56. Müller A, Schaefer T, Linke W, Tudyka T, Gottwald M, Klaus W, Dhein S. (1997). Actions of the antiarrhythmic peptide AAP10 on intercellular coupling. Naunyn Schmiedebergs Arch Pharmacol. 356(1):76-82. doi: 10.1007/pl00005031.
57. Münster PN, Weingart R. (1993). Effects of phorbol ester on gap junctions of neonatal rat heart cells. Pflugers Arch. 423(3-4):181-8. doi: 10.1007/BF00374392.
58. Niggli E, Rüdisüli A, Maurer P, Weingart R. (1989). Effects of general anesthetics on current flow across membranes in guinea pig myocytes. Am J Physiol 256(2 Pt 1):C273-81. doi: 10.1152/ajpcell.1989.256.2.C273.
59. Nguyen TP, Xie Y, Garfinkel A, Qu Z, Weiss JN. (2012). Arrhythmogenic consequences of myofibroblast-myocyte coupling. Cardiovasc Res 93(2):242-51. doi: 10.1093/cvr/cvr292.
60. Noma A, Tsuboi N. (1987). Dependence of junctional conductance on proton, calcium and magnesium ions in cardiac paired cells of guinea-pig. J Physiol. 382:193-211. doi: 10.1113/jphysiol.1987.sp016363.
61. Ortega FA, Grandi E, Krogh-Madsen T, Christini DJ. 2018. Applications of Dynamic Clamp to Cardiac Arrhythmia Research: Role in Drug Target Discovery and Safety Pharmacology Testing. Front Physiol. 8:1099. doi: 10.3389/fphys.2017.01099. eCollection 2017.
62. Patel YA, George A, Dorval AD, White JA, Christini DJ, Butera RJ. 2017. Hard real-time closed-loop electrophysiology with the Real-Time eXperiment Interface (RTXI). PLoS Comput Biol; 13(5):e1005430. doi: 10.1371/journal.pcbi.1005430.
63. Pollard AE, Cascio WE, Fast VG, Knisley SB. (2002). Modulation of triggered activity by uncoupling in the ischemic border. A model study with phase 1b-like conditions. Cardiovasc Res 56(3):381-92. doi: 10.1016/s0008-6363(02)00598-9.
64. Quach B, Krogh-Madsen T, Entcheva E, Christini DJ. 2018. Light-Activated Dynamic Clamp Using iPSC-Derived Cardiomyocytes. Biophys J ;115(11):2206-2217. doi: 10.1016/j.bpj.2018.10.018.
65. Ravagli E, Bucchi A, Bartolucci C, Paina M, Baruscotti M, DiFrancesco D, Severi S. 2016. Cell-specific Dynamic Clamp analysis of the role of funny If current in cardiac pacemaking. Prog Biophys Mol Biol; 120(1-3):50-66. doi: 10.1016/j.pbiomolbio.2015.12.004.
66. Rook MB, van Ginneken AC, de Jonge B, el Aoumari A, Gros D, Jongsma HJ. (1992). Differences in gap junction channels between cardiac myocytes, fibroblasts, and heterologous pairs. Am J Physiol. 263(5 Pt 1):C959-77. doi: 10.1152/ajpcell.1992.263.5.C959.
67. Rüdisüli A, Weingart R. (1989). Electrical properties of gap junction channels in guinea-pig ventricular cell pairs revealed by exposure to heptanol. Pflugers Arch 415(1):12-21. doi: 10.1007/BF00373136.
68. Sands GB, Ashton JL, Trew ML, Baddeley D, Walton RD, Benoist D, Efimov IR, Smith NP, Bernus O, Smaill BH. (2022). It's clearly the heart! Optical transparency, cardiac tissue imaging, and computer modelling. Prog Biophys Mol Biol 168:18-32. doi: 10.1016/j.pbiomolbio.2021.06.005.
69. Shibukawa, Y, Chilton EL, MacCannell KA, Clark RB, Giles WR. (2005). K1 currents activated by depolarization in cardiac fibroblasts. Biophys. J. 88:3924–3935.
70. Schmilinsky-Fluri G, Rüdisüli A, Willi M, Rohr S, Weingart R. (1990). Effects of arachidonic acid on the gap junctions of neonatal rat heart cells. Pflugers Arch. 417(2):149-56. doi: 10.1007/BF00370692.
71. Schmilinsky-Fluri G, Valiunas V, Willi M, Weingart R. (1997). Modulation of cardiac gap junctions: the mode of action of arachidonic acid. J Mol Cell Cardiol. 29(6):1703-13. doi: 10.1006/jmcc.1997.0409.
72. Sheu SS, Fozzard HA. (1985). Na/Ca exchange in the intact cardiac cell. J. Gen. Physiol. 85: 476-478
73. Spitzer KW, Pollard AE, Yang L, Zaniboni M, Cordeiro JM, Huelsing DJ. (2006). Cell-to-cell electrical interactions during early and late repolarization. J Cardiovasc Electrophysiol 17 Suppl 1:S8-S14. doi: 10.1111/j.1540-8167.2006.00379.x.
74. Spray DC, Harris AL, Bennett MV. (1981). Equilibrium properties of a voltage-dependent junctional conductance J Gen Physiol 77(1):77-93. doi: 10.1085/jgp.77.1.77.
75. Sugiura H, Joyner RW. (1992). Action potential conduction between guinea pig ventricular cells can be modulated by calcium current. Am J Physiol. 263(5 Pt 2):H1591-604. doi: 10.1152/ajpheart.1992.263.5.H1591.
76. Tan RC, Joyner RW. (1990). Electrotonic influences on action potentials from isolated ventricular cells. Circ Res. 67(5):1071-81. doi: 10.1161/01.res.67.5.1071.
77. ten Tusscher KH, Panfilov AV. (2006). Alternans and spiral breakup in a human ventricular

tissue model. Am J Physiol Heart Circ Physiol; 291:H1088–100.

1. Terrar DA, Victory JG. (1988). Influence of halothane on electrical coupling in cell pairs isolated from guinea-pig ventricle. Br J Pharmacol 94(2):509-14. doi: 10.1111/j.1476-5381.1988.tb11554.x.
2. Tsuboi N, Kodama I. (1986). Calcium and potassium currents in cardiac cells. Jpn. Heart J. 27, SuppZ. 1: 31-50.
3. Verheule S, Van Kempen MJ, Welscher PHT, Kwak BR, Jongsma HJ. (1997). Characterization of gap junction channels in adult rabbit atrial and ventricular myocardium. Circ Res. 80(5):673-81. doi: 10.1161/01.res.80.5.673.
4. Wagner MB, Wang YG, Kumar R, Golod DA, Goolsby WN, Joyner RW. (2000). Measurements of calcium transients in ventricular cells during discontinuous action potential conduction. Am J Physiol Heart Circ Physiol. 278(2):H444-51. doi: 10.1152/ajpheart.2000.278.2.H444.
5. Wang L, Xu Z, Huang X, Bai X, Wang Z, Yang R, Deng Q, Gao H. 2025. Disrupting the interaction between connexin 43 and calmodulin restores gap junction function and mitigates reperfusion arrhythmias. Sci Rep; 15(1):34716. doi: 10.1038/s41598-025-18366-3.
6. Wei N, Mori Y. 2025. Role of ionic electrodiffusion and ephaptic coupling in cardiac dynamics. Biophys J; 124(18):3060-3074. doi: 10.1016/j.bpj.2025.08.008.
7. Wei N, Tolkacheva EG. 2025. The role of ephaptic coupling and gap junctional coupling in modulating the initiation and dynamics of reentrant arrhythmias. PLoS One; 20(8):e0330016. doi: 10.1371/journal.pone.0330016.
8. Weingart R. (1986). Electrical properties of the nexal membrane studied in rat ventricular cell pairs. J Physiol. 370:267-84. doi: 10.1113/jphysiol.1986.sp015934.
9. Weingart R, Maurer P. (1988). Action potential transfer in cell pairs isolated from adult rat and guinea pig ventricles. Circ Res. 63(1):72-80. doi: 10.1161/01.res.63.1.72.
10. Weiss DL, Ifland M, Sachse FB, Seemann G, Dössel O. (2009). Modeling of cardiac ischemia in human myocytes and tissue including spatiotemporal electrophysiological variations. Biomed Tech (Berl) 54(3):107-25. doi: 10.1515/BMT.2009.016.
11. White RL, Spray DC, Campos de Carvalho AC, Wittenberg BA, Bennett MV. (1985). Some electrical and pharmacological properties of gap junctions between adult ventricular myocytes. Am J Physiol. 249(5 Pt 1):C447-55. doi: 10.1152/ajpcell.1985.249.5.C447.
12. Wilders R, Kumar R, Joyner RW, Jongsma HJ, Verheijck EE, Golod D, van Ginneken AC, Goolsby WN. (1996). Action potential conduction between a ventricular cell model and an isolated ventricular cell. Biophys J. 70(1):281-95. doi: 10.1016/S0006-3495(96)79569-3.
13. Wilders R, Verheijck EE, Joyner RW, Golod DA, Kumar R, van Ginneken AC, Bouman LN, Jongsma HJ. (1999). Effects of ischemia on discontinuous action potential conduction in hybrid pairs of ventricular cells. Circulation; 99(12):1623-9. doi: 10.1161/01.cir.99.12.1623.
14. Whittaker DG, Clerx M, Lei CL, Christini DJ, Mirams GR. 2020. Calibration of ionic and cellular cardiac electrophysiology models. Wiley Interdiscip Rev Syst Biol Med; 12(4):e1482. doi: 10.1002/wsbm.1482.
15. Wittenberg BA, White RL, Ginzberg RD, Spray DC. (1986). Effect of calcium on the dissociation of the mature rat heart into individual and paired myocytes: electrical properties of cell pairs. Circ Res. 59(2):143-50. doi: 10.1161/01.res.59.2.143.
16. Wu X, Payne LB, Gourdie RG. 2025. Gap junctional and ephaptic coupling in cardiac electrical propagation: homocellular and heterocellular perspectives. J Physiol; doi: 10.1113/JP287358.
17. Xie Y, Garfinkel A, Camelliti P, Kohl P, Weiss JN, Qu Z. (2009). Effects of fibroblast-myocyte coupling on cardiac conduction and vulnerability to reentry: A computational study. Heart Rhythm 6(11):1641-9. doi: 10.1016/j.hrthm.2009.08.003.
18. Xie Y, Garfinkel A, Weiss JN, Qu Z. (2009). Cardiac alternans induced by fibroblast-myocyte coupling: mechanistic insights from computational models. Am J Physiol Heart Circ Physiol 297(2):H775-84. doi: 10.1152/ajpheart.00341
19. Zaniboni M, Pollard AE, Yang L, Spitzer KW. (2000). Beat-to-beat repolarization variability in ventricular myocytes and its suppression by electrical coupling. Am J Physiol Heart Circ Physiol 278(3):H677-87. doi: 10.1152/ajpheart.2000.278.3.H677.
20. Zaniboni M, Rossini A, Swietach P, Banger N, Spitzer KW, Vaughan-Jones RD. (2003). Proton permeation through the myocardial gap junction. Circ Res. 93(8):726-35. doi: 10.1161/01.RES.0000093986.47383.CE.
21. Zaniboni M, Cacciani F, Groppi M. (2005). Effect of input resistance voltage-dependency on DC estimate of membrane capacitance in cardiac myocytes. Biophys J 89(3):2170-81. doi: 10.1529/biophysj.105.062828.
22. Zaniboni M, Cacciani F, Salvarani N. (2007). Temporal variability of repolarization in rat ventricular myocytes paced with time-varying frequencies. Exp Physiol 92(5):859-69. doi: 10.1113/expphysiol.2007.037986.
23. Zaniboni M, Riva I, Cacciani F, Groppi M. (2010). How different two almost identical action potentials can be: a model study on cardiac repolarization. Math Biosci 228(1):56-70. doi: 10.1016/j.mbs.2010.08.007.
24. Zaniboni M. (2011). 3D current-voltage-time surfaces unveil critical repolarization differences underlying similar cardiac action potentials: A model study. Math Biosci 233(2):98-110. doi: 10.1016/j.mbs.2011.06.008.
25. Zaniboni M. (2012). Late phase of repolarization is autoregenerative and scales linearly with action potential duration in mammals ventricular myocytes: a model study. IEEE Trans Biomed Eng 59(1):226-33. doi: 10.1109/TBME.2011.2170987.
26. Zaniboni M. (2012). Heterogeneity of intrinsic repolarization properties within the human heart: new insights from simulated three-dimensional current surfaces. IEEE Trans Biomed Eng (8):2372-80. doi: 10.1109/TBME.2012.2204880.
27. Zaniboni M. (2024). In silico analysis of ventricular action potential with a current-voltage-time representation: Thresholds, membrane resistance, repolarization reserve. Physiol Rep 12(21):e70085. doi: 10.14814/phy2.70085.
28. Zeisberg EM, Kalluri R. (2010). Origins of cardiac fibroblasts. Circ Res 26;107(11):1304-12. doi: 10.1161/CIRCRESAHA.110.231910.

**FIGURE LEGENDS**

**Figure 1**. **Cell pairs and their equivalent circuit.** After enzymatic dispersion, cell pairs can be found (A) (from Kieval et al 1992) and braught simultaneously in patch clamp whole cell configuration (B, the shades of the two patch pipettes are visibile) (from Zaniboni et al 2006). White arrows points to the gap junctional region. The equivalent electrical circuit of the cell pair forms the so-called *delta circuit* (C), which appears simplified (D) when considered in steady state conditions.

**Figure 2**. **Pairs configurations.** In the yeald of enzymatically dispersed cells, cell pairs can be found, which are coupled by gap junctions longitudinally (side-by-side, on the left) (from Spitzer et al 2006) or at their extremes (end-to-end, on the right) (from Zaniboni et al 2006).

**Figure 3**. **The coupling-clamp technique.** Two separate cells are brought simultaneously in patch clamp whole cell configuration (left panel, Zaniboni, unpublished results) and the membrane potentials recorded by the patch amplifiers are sent to additional amplifiers (right panel) to compute the voltage difference (ΔV_m_). The outputs are sent to voltage-current converters that supply equal and opposite current into the two cells, exactly as they were directly connected with an electrical resistance R_j_, which can thus be chosen arbitrarily by varying the gain of the amplifiers. The equivalent electrical circuit of two coupling-clamped cells is thus identical to that reported in figure 1 (modified by Huelsing et al 2000).

**Figure 4**. **Measure of R_j_ with double current clamp.** A constant current I_1_, passed through the first electrode into cell 1, produced a voltage deflection ΔV_11_ in the injected cell 1 and ΔV_21_ in the follower cell 2. Similarly, a constant current I_2_, injected into cell 2, produced a voltage deflection ΔV_22_ in the injected cell 2 and ΔV_12_ in the cell 1 (from Kameyama 1983). By applying Ohm’s and Kirchhoff’s laws to these data, the values of R_1_, R_2_, and R_j_ can be derived.

**Figure 5**. **The electrical load.** Mathematical model referred to the behavior of the electrical circuit of figure 1 D (steady state). The intrinsic resting potential of cell 1 is E_1_ = -55 mV, that of cell 2 is E_2_ = -80 mV, thus V_1_ = E_1_ and V_2_ = E_2_ when cells are uncoupled (R_j_ = ∞). The steady state response of V_1_ and V_2_ as electrical coupling between cells increases (R_j_ → 0) is reported in the case when R_1_ = R_2_ (A) and when R_1_ = 10 R_2_ (B). Note that the electrical load is symmetrical (and opposite) in the case of identical membrane resistances, whereas is much higher for the cell with the greater membrane resistance in the case of different resistances (modified from Spitzer et al 1997)

**Figure 6. Action potential transfer in a real guinea pig ventricular cell pair.** A guinea pig ventricular cell pair was perfused with 75 μM of the gap junctional uncoupler β-glycyrrhetinic acid (GA), the junctional resistance continuously measured with double-current clamp protocol described in figure 4, and only cell 1 paced at 1 Hz. (left) *Continuous conduction.* At start, when GA still did not have any effect (low R_j_), the threshold was reached almost simultaneously in cell 1 (black) and cell 2 (red) and AP was conducted between the two with virtually no delay. (middle) *Discontinuous conduction.* When GA caused a significant rise in R_j_, a measurable delay was found between the rising times of the two APs. (right) For longer exposure to GA, R_j_ increased further and AP conduction failed. Note the depolarizing notch in AP1 (blue arrow), due to the depolarizing source of AP2 upstroke. (modified from Zaniboni et al 2003)

**Figure 7. R_j_ physiological ranges.** Indicative ranges covered by R_j_ in three mammalian species in control conditions. See text for description.

**Figure 8. Trans-junctional current behaves ohmically in ventricular pairs.** Voltage pulses of variable amplitude and polarity were applied to one cell of a pair to determine the relationship between junctional current I_j_ and trans-junctional voltage V_j_. Control (o) and after exposure to 2 μM strophantidin (Δ), a gap junctional uncoupler. From linear regression analysis the control value of R_j_ was 19 MΩ, that after strophantidin was 295 MΩ. (from Maurer and Weingart 1987)

**Figure 9. Role of calcium current in AP conduction.** (Top) In a coupling clamped (Rj = 143 MΩ) pair of guinea pig ventricular myocytes, cell 1 was paced and fired an AP (solid line), which propagated to cell 2 (dotted line) with a delay of 6 ms. The perfusion of 1 μM Nifedipine caused an increase of the delay to 9 ms and an increase in the early repolarization rate of AP1. (Bottom) Similar effects were obtained during a premature stimulation (from basic cycle lenght of 500 ms to 195 ms) of cell 1 in a pair of coupling clamped (Rj = 83 MΩ) myocytes. The first on the left is the last conditioning AP at BCL = 500 ms, the second on the right is the first AP elicited prematurely. Because the incomplete removal of inactivation, I_CaL_ is reduced during the premature beat, thus causing less depolarizing junctional current, increasing the delay between the two APs, and dramatically increasing early repolarization rate in cell 1. (from Sugiura and Joyner 1992)

**Figure 10. Role of transient-outward potassium current in AP conduction.** (A) AP conduction in a pair of rabbit right ventricular myocytes coupling clamped at their critical R_j_. Leader AP is continuous line, follower dotted line. (B) When I_TO_ was inhibited with 4-aminopyridine and R_j_ kept at the critical value of the control condition, this led to shortening of the delay. (C) R_j_ was gradually increased until the new critical value in the presence of the I_TO_ blocker was found, significantly increased respect to control. (from Huelsing et al 2001)

**Figure 11. Symmetric vs asymmetric AP conduction.** (A) In a pair of cells with similar input resistances (22 vs 21 MΩ), at first only cell 1 was paced (top) and, in turn, only cell 2 was paced (bottom) for the same junctional R_j_ (300 MΩ). AP conduction took place in both directions with very similar delay. (B) In a pair of cells with cell 1 having a much higher input resistance than cell 2 (73 vs 30 MΩ), and for the same junctional R_j_ (500 MΩ)_,_ AP conduction failed when cell 1 was paced and succeeded when cell 2 was paced. (from Joyner et al 1991)

**Figure 12. Asymmetry in APD changes.** By increasing or decreasing the maximum conductance of I_kr_ in the Luo and Rudy AP model, two intrinsically short (S) and long (L) AP were obtained. When electrical coupling between the two was simulated, they reached a common coupled configuration where the shortening of the longest AP was greater than the prolongation of the shorter. (from Zaniboni et al 2000)

**Figure 13. Asymmetric APD changes in ventricular-Purkinje pairs.** In a pair of simultaneously paced ventricular-Purkinje myocytes (V and P) with shorter and longer intrinsic AP waveforms respectively, coupling clamping with a R_j_ = 50 MΩ led to a common coupled waveform (♦), whose APD was shorter than both intrinsic APDs. (from Huelsing et al 1999)

**Figure 14. Suppression and facilitation of EADs by coupling with resting cell.** (A) A real Purkinje cell was coupling clamped with an RC circuit with the same passive electrical properties of a ventricular myocyte (membrane potential V_RC_ of the RC circuit can be arbitrarily set). (B and C) When V_RC_ was well polarized, isoproterenol-induced EADs in the Purkinje cell were suppressed by electrotonic coupling with progressively smaller R_j_ values. (D) As V_RC_ became less polarized, electrical coupling did not prevent EADs formation. (from Huelsing et al 2000)

**Figure 15. Electrotonic suppression of beat-to-beat variability of APD.** Successive APs were recorded from two different isolated ventricular myocyts, one with intrinsically shorter APD (top), and the other with intrinsically longer APD (bottom), both showing intrinsic beat-to-beat APD variability. When the two cells were coupled with a R_j_ = 100 MΩ, they reached a common coupled waveform which showed a significantly smaller beat-to-beat APD variability. (from Zaniboni et al 2000)

**Figure 16. Response to high pacing frequency.** (A-C) Simulated results of coupling two Beeler and Reuter models, only one paced, and (D) a Beeler and Reuter model with a passive RC circuit (stimulation patterns are indicated by asterisks and numbers). In all simulations the pacing CL was switched from 1000 ms to shorter values at beat 1; coupling resistance is reported in figure for each simulation. (A) At this level of electrical coupling, the paced cell (CL = 200 ms) showed 2:1 APD alternans, whereas the following cell only showed sub-threshold electrotonic depolarizations. (B) The paced cell (CL = 200 ms) showed APD 1:1 alternans, with following cell showing sub-threshold electrotonic depolarizations. (C) Paced cells showed 4:3 activation and following cell 4:1 activation. (D) Transition from CL = 500 ms to CL = 150 ms led to 1:1 APD alternans in the paced cell when uncoupled from the RC model, regular 1:1 beating when coupled. (from Tan and Joyner 1990)

**Figure 17. Ventricular myocyte-fibroblast coupling.** (A) Simulated coupling of one, two, and 4 fibroblasts embedded with the four ionic currents described in the text (active fibroblst) with a ventricular myocyte, and with a junctional resistance R_j_ = 333 MΩ. (B) Simulated coupling of a ventricular myocyte with two active fibroblasts with junctional resistances R_j_ = 1000, 500, and 333 MΩ. (modified from Mac Cannell et al 2007)

**Figure 18. Ventricular myocyte-fibroblast coupling can promote APD alternans.** The Luo and Rudy model of a myocyte was coupled with a different number of fibroblasts at different BCLs. (A) BCLs at which APD alternans occurred as a function of the number of fibroblasts coupled to a myocyte. (B) bifurcation diagram showing APD vs BCL for a myocyte coupled to 8 fibroblasts. (C) AP sequence for BCL = 500 ms. (resting membrane potential of the fibroblast was -50 mV, inter-fibroblast R_j_ = 5000 MΩ, fibroblast-myocyte R_j_ = 125 MΩ). (modified from Xie et al 2009 B)

**Figure 19. The early I_to_-like component of the junctional current promotes EADs in myocyte-myofibroblast pairs.** A real patch clamped myocyte, paced at BCL = 6s, was exposed to oxidative stress to induce EADs (star), which were suppressed at BCL = 1 s (not shown). When the myocyte was coupled to a virtual fibroblast (C_m_=6.3 pF, E_r_ = -25 mV, R_j_ = 3.3 GΩ) EADs reappears (star, left upper trace). The junctional current (left lower trace) consisted of an early transient outward I_to_-like component followed by a sustained component. When coupling was allowed only during the first 100 ms of the AP cycle, the EAD persisted (middle upper trace), whereas when coupling was allowed only during the last 900 ms of the cycle, the EAD was suppressed (right upper trace). (modified from Nguyen et al 2012)
